# Robotic Materials With Bioinspired Microstructures for High Sensitivity and Fast Actuation

**DOI:** 10.1002/advs.202509739

**Published:** 2025-09-25

**Authors:** Sakshi Sakshi, Rohit Pratyush Behera, Hongyu Zhou, Yifan Wang, Hortense Le Ferrand

**Affiliations:** ^1^ School of Mechanical and Aerospace Engineering Nanyang Technological University Singapore 637460 Singapore; ^2^ School of Material Science and Engineering Nanyang Technological University Singapore 639798 Singapore

**Keywords:** actuation, bioinspiration, computation, functional smart materials, microstructures, robotic materials, sensing

## Abstract

Functional soft materials with sensing and actuation capabilities enable the creation of autonomous devices and structures. Taking inspiration from natural microstructures of living organisms, a new generation of robotic materials that sense, compute, and actuate is being developed. This manuscript reviews how the integration of bioinspired microstructures into soft materials contributes to enhancing their performance for pressure sensing, actuation, and computation. The design principles for developing such microstructures are outlined, along with discussions of the fabrication strategies. Performance maps are drawn from literature data to allow comparisons of capabilities and to determine trends. Finally, the emerging approaches to embed computation with sensing and actuating into a single robotic material are presented. Overall, this review demonstrates that leveraging bioinspired microstructures into synthetic and functional systems can unlock new material properties that could be deployed in larger autonomous and self‐adaptable structures. In the context of sustainable development, relying on microstructure for performance allows the use of new feedstocks while achieving desired functionalities.

## Graphical abstract

In the review paper, design rationale and approaches for bioinspired sensors and actuators in robotics applications are presented. These bioinspired microstructure strategies implemented in both can improve the performance in several ways. Also, recent ideas and innovations that embed robotic materials with logic and computation with it are part of the discussion.

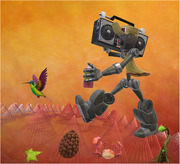

## 1 Introduction

Robotic materials are smart materials that can sense, compute, and actuate, in order to adapt to the changes of their environment, opening the ability to shape an autonomous world around us.^[^ ^1^ ^]^ The incorporation of soft materials into robotic systems for sensing, actuation, energy storage, and interfaces drastically differs from traditional robotics. In traditional systems, materials are typically stiff and provide the mechanical support, while joints and additional modules allow flexibility alongside fast and adaptable sensing and actuation.^[^ ^2^, ^3^ ^]^ Indeed, traditional robots rely on voltage‐based signal transmission to processors, algorithmic decision‐making, and motor‐driven actuators connected by hinges or joints to operate. The complexity of this assembly and the heavyweight restrict their applications. For example, in scenarios where bulky, complex, and rigid control systems are impractical, there is a need for untethered soft and flexible robots that can move and make decisions autonomously. In addition to challenges in applications, parts of traditional stiff robots utilise raw feedstocks like heavy metals, which have a high environmental and ethical cost. To palliate this issue, the use of functional soft materials in robots started about a decade ago and has rapidly expanded into a large variety of devices and structures that are always more autonomous, adaptable, and biomimetic. For making soft robots adaptable to their environment, pressure sensors and actuators are particularly key components as they allow accurate and precise communication and action. Integrating signal transmission and processing directly into smart robotic materials capable of autonomous sensing and actuation is a promising solution to reduce the need for heavy metals, facilitate communication, while improving energy efficiency, versatility, and timely response. These robotic materials, which are soft, flexible, and capable of sensing and actuation, could therefore enable the next generation of robots and other autonomous systems.

**Figure 1:**
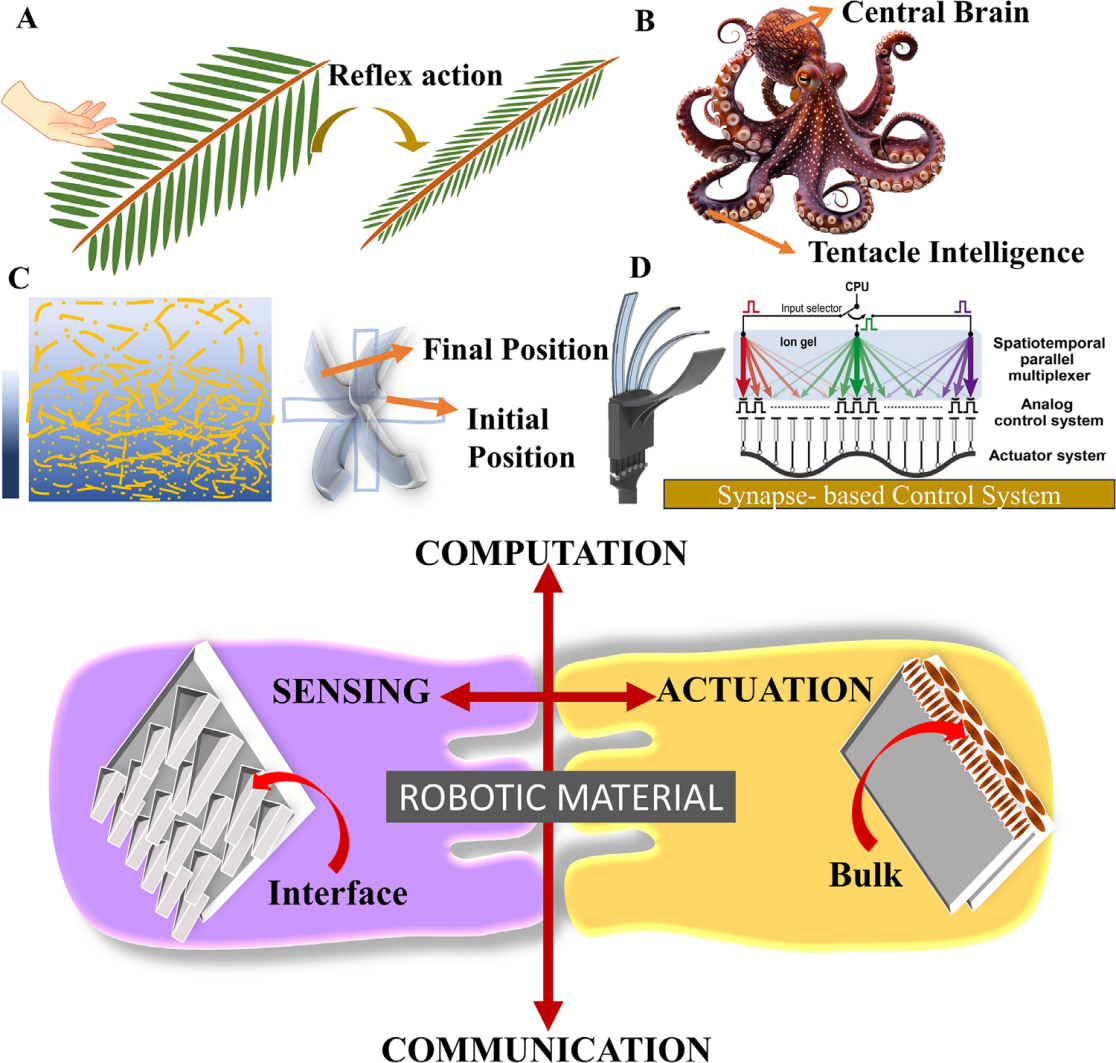
Bioinspiration for Robotic Materials **A)** Reflex Action of *Mimosa pudica* upon touch. **B)** Neural control in the mini‐brains in octopus arms. Reproduced with permissions from Vecteezy.com. **C)** Anisotropic hygroscopic swelling and mechanical stability creating plant‐inspired actuation in soft robotics material. **D)** Human‐like finger coordination using synapse‐based control system. Reproduced under terms of the CC‐BY license.^[^ ^7^ ^]^ Copyright 2023, Dong Gue Roe et al, published by Springer Nature. **E)** Schematics showing tight integration of sensing, actuation, computation, and communication into robotic materials facilitated by bioinspired microstructures.

To unfold the potential of robotic materials, it is essential to look at the biological systems that inspire them. Indeed, these materials take their inspiration from living organisms that typically respond to stimuli such as pressure, humidity, light, etc. to achieve complex, self‐adaptive behaviors. State‐of‐the‐art sources of inspiration range from the reflex action of *Mimosa pudica* to the distributed neural control in the mini‐brains in octopus arms,^[^ ^4^ ^]^ that demonstrate distributed sensing and localized actuation across multiple length scales (Figure **1**A,B). Biological systems are therefore fascinating blueprints for robotic materials as sensing, computation, and actuation are interconnected and interdependent. In more detail, biological sensing is achieved through specialized cells that transduce environmental signals into biochemical or electrical responses, such as the mechanoreceptors on our skin that respond to pressure. Biological actuators convert energy into mechanical motion through muscle contractions and extensions in response to electrical signals from the nerves.^[^ ^5^ ^]^ While living cells are necessary to allow these functions, synthetic systems using complex electrical signals and multiphysics mechanisms could emulate natural sensing and actuation in bioinspired systems.^[^ ^6^ ^]^ For example, complex neurone‐like spike signals have been used to create robotic hands for human‐like finger coordination,^[^ ^7^ ^]^ or anisotropic hygroscopic swelling and mechanical stability have been used for creating plant‐inspired actuation (Figure 1C,D).^[^ ^8^ ^]^ Inspiration from living organisms is, therefore a promising avenue to create robotic materials for complex autonomous synthetic structures.

Bioinspiration is the design approach that consists of transposing the mechanisms from a biological system into a synthetic counterpart. While bioinspiration can be related to transposing the composition or the shape, taking inspiration from microstructures, which define the way materials are organized at the micro and sub‐micro scales has shown promising capabilities in realizing high‐performance, multifunctional, and sustainable materials.^[^ ^9^, ^10^ ^]^ For example, many studies report on how microstructuring can enhance sensitivity in dielectrics and electrodes,^[^ ^11^, ^12^, ^13^ ^]^ with up to a 30‐fold improvement.^[^ ^14^ ^]^ Because pressure sensing primarily depends on changes at interfaces due to stress concentration or redistribution, microstructuring in sensors usually involves introducing surface‐level micropatterns, such as micropyramids,^[^ ^15^ ^]^ micropillars^[^ ^16^ ^]^ or microdomes.^[^ ^17^ ^]^ These patterns improve sensitivity, enable better stress localization, and enhance the overall performance of the sensor. In actuators, microstructures with different alignments in plants have been shown to store energy which is released upon stimulus, for example, for seed and spore dispersal,^[^ ^18^ ^]^ self‐burying,^[^ ^19^ ^]^ or to capture insects.^[^ ^20^ ^]^ Using bioinspired microstructures for making sensors and actuators can therefore be one way to create robotic materials with higher sensitivity and accuracy in response to one or multiple stimuli across a broad signal range, along with fast, controllable, and versatile response. Many excellent reviews have discussed integration of sensing, actuation and computation in soft robotic materials,^[^ ^1^, ^6^ ^]^ how bioinspired microstructure can enhance properties,^[^ ^21^ ^]^ and other benefits of bioinspiration for robotics.^[^ ^22^, ^23^ ^]^ However, a review on how bioinspired microstructures can provide high‐performing pressure sensors, actuators, and integrate them into robotic materials is still needed.

This review therefore tackles the role of bioinspired microstructures in enhancing performance for sensing, actuation, and their tight integration into robotic materials (Figure 1E). Due to the large extent of the topic, this review does not aim at being comprehensive, but at summarizing the key developments and trends reported in recent literature. First, bioinspired microstructures for pressure sensors, their design strategies, and fabrication methods are reviewed and discussed, and the performance of each design is compared in performance maps. Second, bioinspired microstructural designs for soft actuators, their fabrication methods, and performance are also reviewed, discussed, and compared. Finally, recent developments in using bioinspired microstructures for combining sensing, actuation, and computation into one robotic material are presented. This review highlights the advantages of bioinspired microstructures for such functional applications and could be leveraged by researchers to engineer the next generation of autonomous devices and structures.

## 2 Pressure Sensors with Bioinspired Microstructures

Nature has evolved skin through optimizing structural and functional solutions for complex sensing. Human skin is an intelligent biological interface consisting of intricate microstructures like interlocking dermal ridges, spatially distributed mechanoreceptors, and nerve lattices.^[^ ^24^ ^]^ As we move toward seamless sensing capabilities, it becomes important to shift from conventional planar sensors to bioinspired, microstructured interfaces that hold the potential to significantly advance the sensitivity and linearity. Linearity, among various performance metrics, is the foundation for reliable, interpretable, and efficient pressure sensing. Therefore, skin's hierarchical architecture should be regarded not merely as an inspiration but as a foundational strategy in the design of future sensors. Bioinspired microstructures in pressure sensors enhance pressure sensitivity and linearity. The key design principles for incorporating bioinspired microstructures in sensors are first introduced and compared to conventional and non‐structured sensors. Fabrication strategies for making bioinspired microstructures in capacitive and piezoresistive sensors are also detailed. Finally, the performances of the bioinspired microstructured sensors are collected and presented in performance maps and discussed.

### 2.1 Principles of Sensing and Advantages of Bioinspired Microstructures

**Figure 2:**
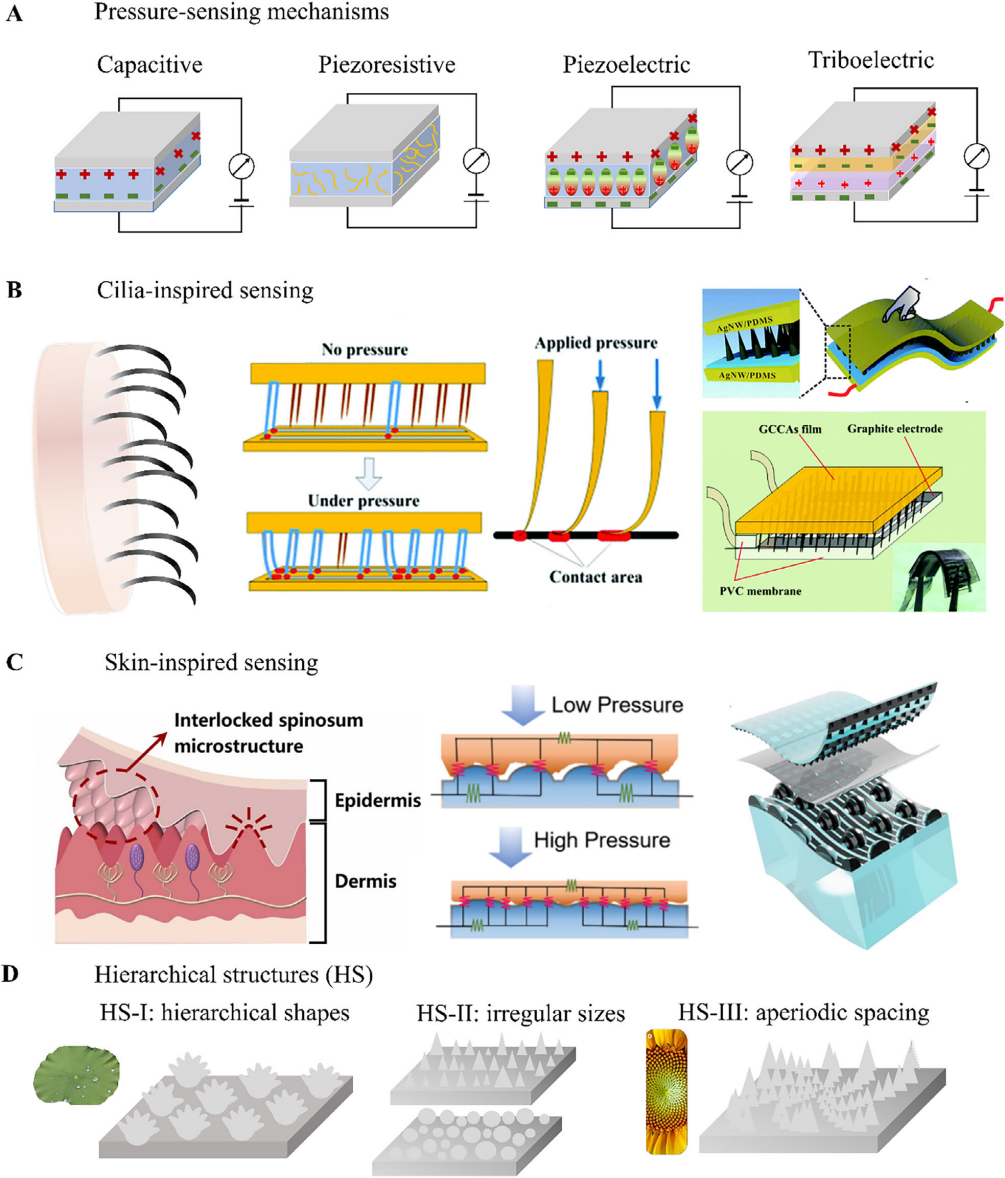
Working mechanisms of pressure sensing materials and bioinspired microstructured sensors. **A)** Schematics illustrating the 4 main types of pressure sensing mechanisms. **B)** Illustration of cilia‐inspired pressure sensing where pressure increases the contact area at the electrode and examples of such structures in capacitive (top) and piezoresistive (bottom) sensors. Reproduced with permissions from ref.[25] Copyright 2018, Royal Society of Chemistry; from ref.[26] Copyright 2019, Royal Society of Chemistry. **C)** Illustrations of human skin with interlocked microstructures, of the principle of a skin‐inspired sensor, and of a skin‐inspired capacitive sensor. Reproduced with permissions from ref.[27] Copyright 2024, Elsevier; ref.[28] Copyright 2024, Elsevier; ref.[29] Copyright 2018, AAAS. **D)** Illustrations of hierarchical microstructures (HS) for augmented sensing with hierarchical shapes (HS‐I), irregular sizes (HS‐II), and aperiodic spacings (HS‐III) mimicking structures found on plant leaves or flowers. Reproduced from A. Marcus, https://digitalprovocations.wordpress.com/2011/11/10/project‐3_2a‐sunflower/; from ref.[29] Copyright 2018, AAAS.

Pressure sensing involves measuring variations of a physical property of a material placed between two electrodes. The nature of the material, which is the pressure sensor, will determine the physical property to record using electrodes. The four major types of pressure sensors are described as well as bioinspired microstructures in pressure sensing and underlying their working principles (**Figure** **2**).

Among the various pressure sensing principles, four major types dominate: capacitive, piezoresistive, piezoelectric, and triboelectric (Figure 2A). They are used to detect force, pressure, and deformation especially for robotic grippers, artificial muscles, e‐skins, and computing devices. Capacitive sensors measure pressure or deformation by detecting changes in capacitance, which is influenced by the dielectric material sandwiched between two conductive electrodes.^[^ ^30^ ^]^ Piezoresistive sensors work due to the change in electrical resistance of a material in response to applied mechanical stress^.[^ ^31^ ^]^ Piezoelectric sensors operate based on the direct piezoelectric effect, where applying mechanical pressure causes an internal shift in charge distribution, leading the buildup of opposite charges on the two surfaces aligned with the material's polarization axis and a change in electrical conductivity.^[^ ^32^ ^]^ Finally, triboelectric sensors work based on the triboelectric effect combined with electrostatic induction. When two materials with different triboelectric properties come into contact and then separate, electrons transfer from one surface to the other. This results in a charge imbalance, and if electrodes are connected to the materials, this imbalance creates a potential difference that drives a current through the external circuit. Triboelectric sensors work on four major modes: contact–separation, lateral sliding, single‐electrode, and freestanding triboelectric‐layer modes.^[^ ^33^ ^]^ These four pressure sensing mechanisms heavily rely on contact area and distance between electrodes, which can be tailored using microstructured materials. Microstructures such as domes, pyramids, or pillars localize and guide deformation, which reduces lags while loading and unloading, also known as hysteresis.

Taking inspiration from biological systems like cilia and human skin, researchers and engineers have introduced microstructures that augment the sensing properties based on these four sensing mechanisms. Microstructures help to reduce the hysteresis effects, widening the linear range of the pressure and enhancing sensitivity.^[^ ^34^ ^]^ When pressure is applied to a sensor with embedded air gaps due to microstructuring, these air‐filled regions can compress significantly before the material itself deforms. Thus, microstructuring provides unique ways to introduce air gaps in the layer and to enhance the sensing range. For example, applying pressure on sensors with cilia‐inspired microstructures, leads to the continuous expulsion of trapped air within the dielectric layer, thereby enhancing contact and signal generation. Cilia‐like microstructures enhance sensor performance primarily through mechanical deformation of the cilia and increased contact area upon pressure (Figure 2B).^[^ ^25^ ^]^ This deformation can transpose slight mechanical stimuli into amplified structural changes. Flexible capacitive pressure sensors with high sensitivity and a broad detection range can be developed using a hair‐like micro cilia array (MCA) in a dielectric medium.^[^ ^26^ ^]^ Similarly, electronic cilia from graphene‐coated magnetic cilia arrays can be developed for pressure sensing.^[^ ^25^ ^]^ The increase of the contact area between the cilia layer and the electrode layers is very important to the sensitivity of the sensor.

Another common example of bioinspired microstructuring takes inspiration from skin, which has a complex organisation at multiple levels (Figure 2C). Skin contain a wavy structure called interlocked spinosum microstructure, which gives it the ability to deform and recover under varying pressure.^[^ ^29^ ^]^ In addition to this, different mechanoreceptors in the skin can respond to static pressure as well as dynamic forces and vibrations, enabling the detection of subtle stimuli such as light touch, vibration, and stretch.^[^ ^35^ ^]^ When an external pressure is applied, these interlocked features compress and deform, leading to significant variation in contact area and stress concentration.^[^ ^28^ ^]^ This design effectively extends the sensing range, which is a major drawback of single‐layer microstructures. Along with widening the range of pressure, researchers have also developed an interlocking dermal‐epidermal junction in a capacitive sensor for detecting the direction of the force.^[^ ^29^ ^]^ These biologically inspired geometries serve as foundational elements for more complex hierarchical structures (HS) that consist of a flexible flat layer with interlocked protruding features on top (Figure 2D). HS facilitates multiple contact points between the sensing and the electrode layers, further improving the sensing range and linearity of the sensor.^[^ ^36^ ^]^ HS structures are commonly found across the scientific literature, although they may be referred to only as bioinspired microstructures. We have grouped them into three main categories, I, II, and III. Hierarchical structures I (HS‐I) are sensors where the protrusions have a hierarchical shape, such as the micropapillae with several nanofolds found in rose petals,^[^ ^37^, ^38^ ^]^ the hierarchical structure found in lotus leaves with a two‐level roughness,^[^ ^39^, ^40^ ^]^ and numerous other leaves' surface patterns: banana leaf, mimosa,^[^ ^41^ ^]^ *Epipremnum aureum*,^[^ ^42^, ^43^, ^44^ ^]^ reed leaf,^[^ ^45^ ^]^ Gingko leaves,^[^ ^46^ ^]^ and *Calathea zebrine*.^[^ ^47^ ^]^ Hierarchical structures II (HS‐II) are sensors where the protrusions have varying heights and other dimensions.^[^ ^34^ ^]^ Due to the observation that in single‐sized microstructures, an increase in sensitivity comes at the cost of higher hysteresis, small microstructure arrays have been embedded within large lattices to minimize hysteresis induced by interfacial adhesion, while reducing the area density of larger microstructures to enhance sensitivity. Finally, hierarchical structures III (HS‐III) are sensors where the protrusions have irregular spacing. HS‐III can also combine features from HS‐I and HS‐II. For example, the spatial organization of sunflower florets has been used to design the positioning of the pyramids positioned along a phyllotaxis spiral grid with two different aspect ratios of pyramids at the spiral centre (aspect ratio 0.4) and border (aspect ratio 4). It has helped in combining multiple advantages like high sensitivity at the centre, while high capacitance, and fast response time in the outer zone.^[^ ^29^ ^]^

Pressure sensors use materials with dielectric, piezoelectric or other properties, which are assembled and fabricated with hierarchical patterns. Fabrication of these sensors demands specific methods which are described in the following.

### 2.2 Fabrication of Pressure Sensors With Bioinspired Microstructures

**Figure 3:**
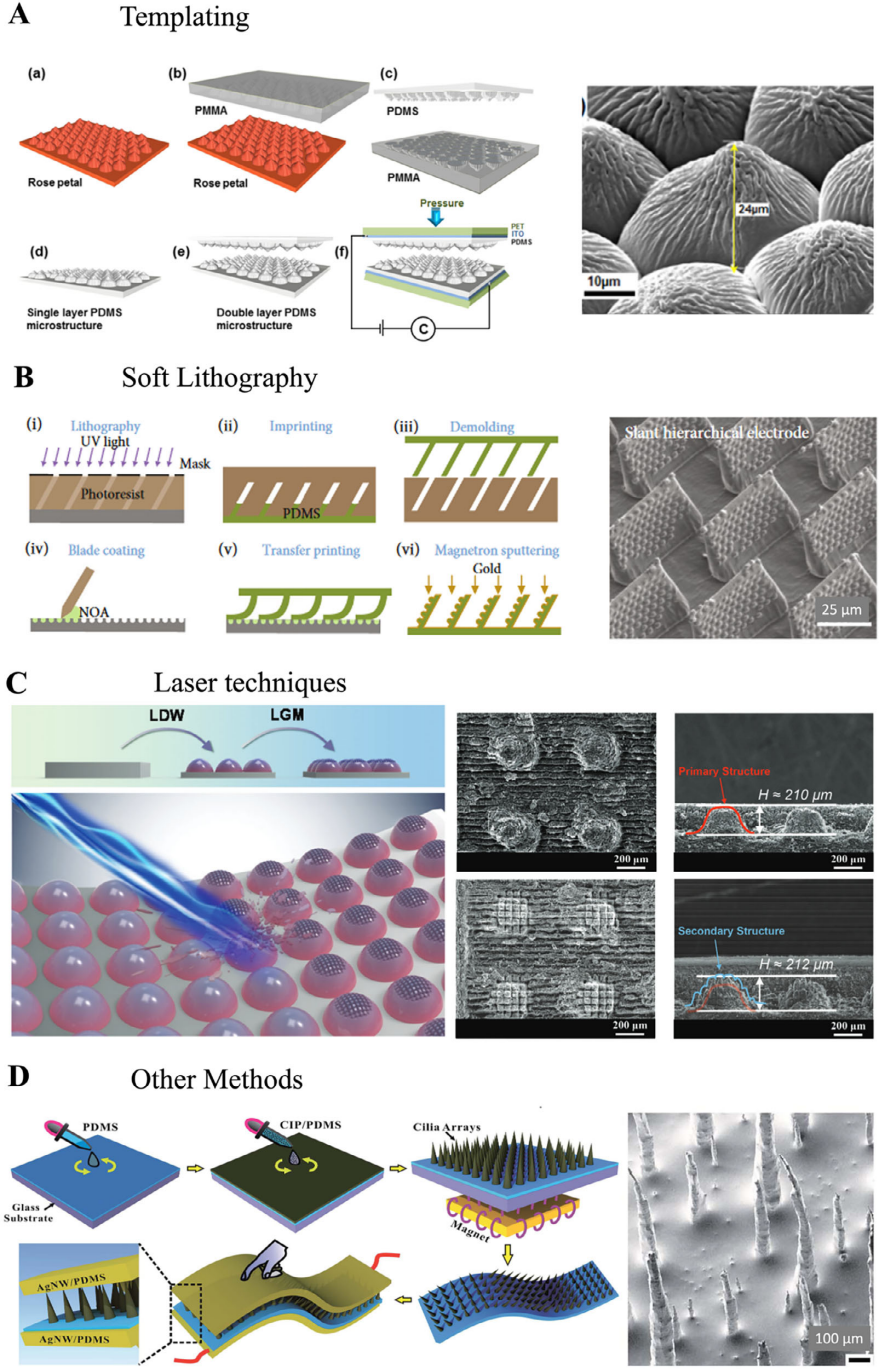
Fabrication approaches for pressure sensors with bioinspired microstructures. **A)** Schematics of the fabrication process of rose petals inspired flexible Polydimethylsiloxane (PDMS) micro/nano microdome‐structures and electron micrographs of the obtained pattern made of Poly (methyl methacrylate) (PMMA). Reproduced with permissions from ref.[48] Copyright 2020, Elsevier. **B)** Manufacturing process of a slanted hierarchical electrode using lithography and an electron micrograph of the structured electrode. Reproduced under terms of the CC‐BY 4.0.^[^ ^49^ ^]^Copyright 2022, Yongsong Luo et al., Published by Exclusive Licensee Science and Technology Review Publishing House. **C)** Illustrations of a laser processing of secondary microstructures on top of primary microstructures and electron micrographs of the primary and secondary structures. Reproduced with permissions from ref.[50] Copyright 2021, Wiley‐VCH. **D)** Schematic of the fabrication process of the capacitive pressure sensor using the Magnetic Cilia Array (MCA) as the dielectric layer and electron micrograph of the MCA. Reproduced with permissions from ref.[26] Copyright 2019,Royal Society of Chemistry.

While lithography has been the most employed fabrication method, new strategies have been developed that combine two or multiple fabrication processes to successfully fabricate hierarchical micro‐nano structures with high reproducibility and scalability (**Figure** **3**).

Templating natural materials offers a simple and easy approach (Figure 3A). It is also cost‐effective and minimizes environmental waste, which makes it the preferred method for sensor microstructuring. To transpose leaves’ or petals’ microstructures, negatives are made by Polyvinyl Alcohol or any other curing polymer like PDMS or Polyvinylidene fluoride onto the natural template. To enhance the replication quality, harder PDMS with a higher Young's modulus than standard PDMS is used, making it suitable for nanoscale pattern replication.^[^ ^30^ ^]^ However, templating only reproduces the feature of the natural materials and does not allow facile customization.

Soft lithography, in turn, is a broader fabrication technique that can create any nano‐ and micro‐structured patterns (Figure 3B). Soft lithography enables precise control over microstructure geometry, size, and spatial arrangement giving an edge in developing more uniform and reproducible microstructured sensors. However, these pressure sensors mostly use etched Si molds^[^ ^29^, ^51^ ^]^ or 3D printed molds^[^ ^52^ ^]^ to develop microstructures. There remains a gap in the fabrication of more complicated structures in terms of implementing advanced lithographic techniques in parallel with capacitive and piezoresistive sensors. In ionotropic sensors that generate signals based on changes in ionic concentration, complex hierarchical structures like gecko feet have been effectively reproduced using oblique lithography techniques.^[^ ^49^ ^]^ The primary structure is formed via imprinting, while tilt angles are tuned by adjusting the photolithography incidence angle, followed by transfer imprinting onto a blade‐coated secondary template in a prefilled UV Adhesive (NOA) using a slant‐scale press. Overall, soft lithography is a highly cost‐effective method with high resolution and large versatility.

Other methods use ultrafast, high‐intensity laser pulses combined with nonlinear absorption. This process causes localized ablation and structural modifications in the underlying material, leading to precise micro‐ and nano‐structures (Figure 3C). Several laser processing parameters, like laser wavelength, pulse duration, laser power, and repetition frequency, can be tuned. Microgrooves have been fabricated using Diode Pumped Solid State (DPSS) laser (355 nm, 0.45 W),^[^ ^53^ ^]^ microdomes (h≈19.5 µm, d≈22.8 µm) and microridges using femtosecond lasers (1030 nm, 780 fs, 5.0 W),^[^ ^53^ ^]^ micropyramids using CO_2_ laser engraved acrylic mould (10.6 µm, 11.25 W).^[^ ^54^ ^]^ A large variety of microstructures with different heights can be obtained by adjusting the laser scanning path.^[^ ^55^ ^]^ However, hierarchical microstructures require fine‐tuning and optimisation of many laser parameters. Feng et. al. have achieved a hierarchical structure in PDMS using a 355 nm femtosecond laser, where a secondary structure is inscribed onto a primary structure.^[^ ^55^ ^]^ Another approach to fabricate these hierarchical structures is to combine different techniques like Laser Direct Writing (LDW) and Laser Gridded Marking (LGM).^[^ ^50^ ^]^ The formation and quality of the secondary microstructures during LGM are influenced by the scanning speed, laser repetition rate, and scanning passes. The scanning speed primarily influenced the morphology, curvature, and feature height, while the laser repetition rate played a crucial role in achieving well‐defined and neat structures.

Finally, micro cilia array (MCA) has been fabricated by spin coating carbonyl iron particles (CIP), PDMS, and a curing agent onto a cured membrane, followed by embedding silver nanowires (AgNWs) for making the electrodes using a magnetic field to induce particle aggregation into cilia‐like patterns (Figure 3D).^[^ ^25^ ^]^ The patterns arise from the combined effects of magnetic force, gravity, and surface tension ensuring the alignment and stabilization of the cilia. Techniques like two‐photon polymerization have also been explored to fabricate cilia‐inspired structure.^[^ ^56^ ^]^

Templating, photolithography, laser ablation techniques, and other combined methods are effective to create pressure sensors with skin‐like, cilia‐like, and other hierarchical microstructures. These sensors exhibit higher performance as compared to others, as reviewed in the next section.

### 2.3 Performance of Bioinspired Microstructured Pressure Sensors

**Figure 4:**
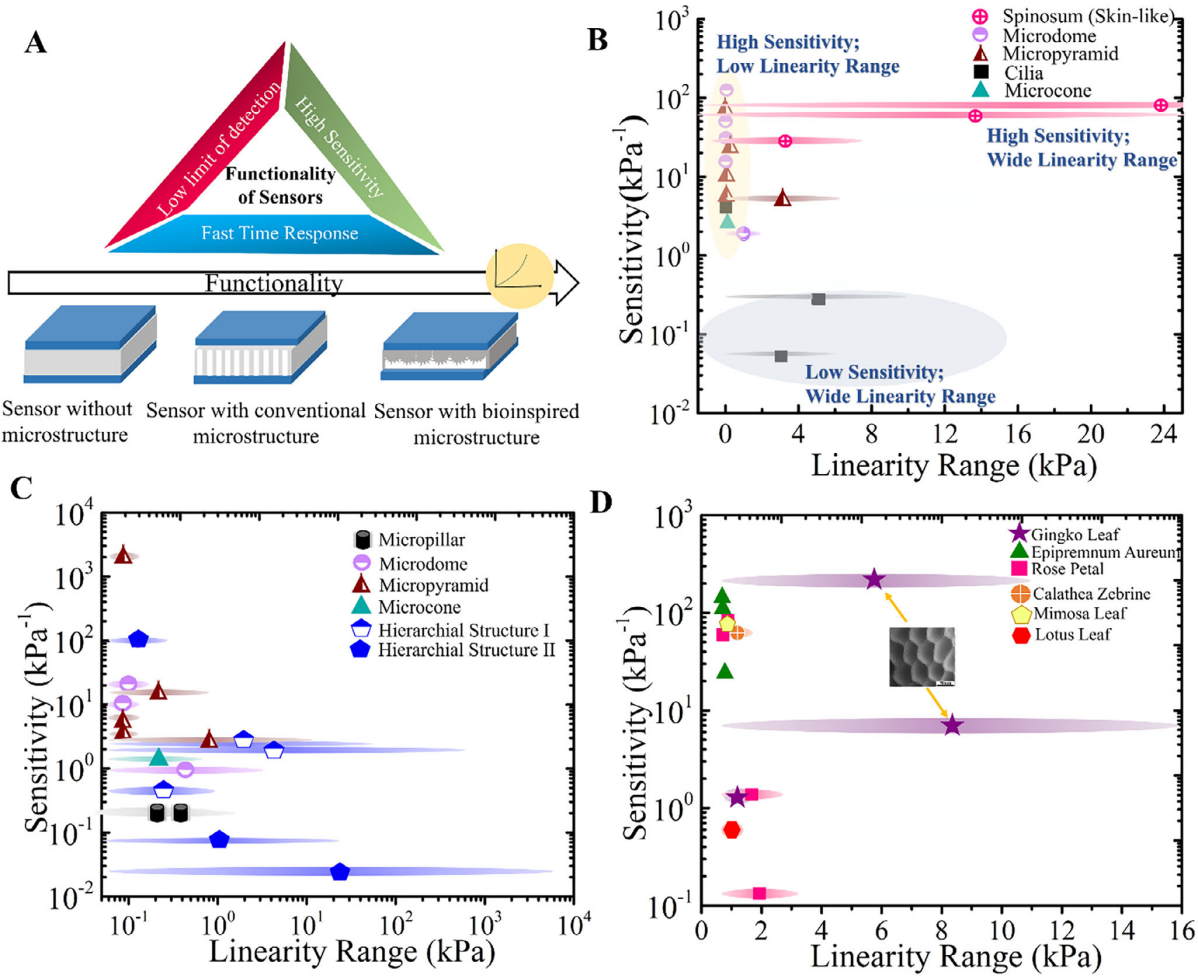
Performance of the bioinspired microstructured pressure sensors. **A)** Increase in functionality and performance of the pressure sensor with addition of bioinspired microstructures. **B)** Sensitivity versus linearity range map of bioinspired microstructure skin‐ (pink) and cilia‐ (black) inspired sensors with other conventional microstructures (micropyramid, microcone, and microdome). **C)** Sensitivity versus linearity range map of conventional microstructures with bioinspired structures HS‐I and HS‐II. **D)** Sensitivity versus linearity range map of bioinspired HS‐I sensors obtained by templating different biological materials (see Table S1, Supporting Information for the raw data).

The performances of conventional and bioinspired microstructured pressure sensors are reviewed and compared in terms of their sensitivity and linearity range (**Figure** **4**, see Table S1, Supporting Information for the comprehensive dataset overview).

Effective pressure sensors have high sensitivity over a broad pressure range, a fast response time, and are durable (Figure 4A). In particular, there is a trade‐off between sensitivity and linearity where an increase in one performance often compromises the other. Bioinspired microstructures help address this challenge. This is illustrated in the performance maps featuring the sensitivity as a function of the linearity range that compare conventional microstructures such as microdomes, microcones, and micropyramids with the bioinspired microstructures: skin and cilia‐inspired (Figure 4B), HS‐I and HS‐II sensors (Figure 4C) and HS‐I structures templated from natural materials (Figure 4D).

Spinosum‐inspired structures exhibit high sensitivity while maintaining the very broad pressure range (Figure 4B). The interlocked structure of skin mimicked using photolithography can show sensitivity as high as 67.1 kPa^−1^ across a wide range of pressures from 0 to 50 kPa,^[^ ^28^ ^]^ whereas spinosum structure fabricated by laser direct writing shows sensitivity of 68.3 kPa^−1^ across the 0–13.5 kPa range.^[^ ^27^ ^]^ However, such an interlocked structure mimicked using an embedding of sea urchin microcapsules (*Helianthus annuus L*. spores) has shown sensitivity up to 24.63 kPa^−1^ under a pressure range of 0–7 kPa, which is also better than other microstructured sensors.^[^ ^57^ ^]^ Overall, sensors with interlocked spinosum‐like structures, regardless of the fabrication techniques employed, outperform sensors with other conventional microstructural designs in terms of high sensitivity across a broad linearity range. Cilia‐inspired microstructures, although exhibiting lower sensitivity as compared to the spinosum ones, can maintain a broader linearity range as compared to conventional microstructures. Cilia‐inspired sensors have shown a linearity range across 0–6 kPa with a sensitivity of only 0.0513 kPa^−1^.^[^ ^56^ ^]^ However, these cilia‐inspired microstructures can also show sensitivity up to 4 kPa^−1^ but can maintain the linearity in the range of 0–0.1 kPa.^[^ ^25^ ^]^ Overall, cilia‐inspired structures can be implemented across a broad pressure range, particularly in applications where lower sensitivity is acceptable.

HS‐I sensors based on templated natural microstructures, demonstrate impressive sensitivity metrics (Figure 4D). For instance, sensors templated from *Ginkgo biloba* leaves exhibit a high sensitivity of up to 164.93 kPa^−1^ across a broad pressure range of 0–10 kPa, making *Ginkgo biloba* leaves one of the most effective natural templates.^[^ ^58^ ^]^ In comparison, rose‐petal templated sensors achieve sensitivities of up to 70 kPa^−1^ across a linear range of only 0–0.5 kPa.^[^ ^59^ ^]^ Similarly, *Calathea zebrina*‐based structures show a sensitivity of 54.31 kPa^−1^ across the same range.^[^ ^47^ ^]^ Sensors templated with mimosa leaves reach up to 50 kPa^−1^ but are constrained to an even narrower linear range of 0–0.07 kPa.^[^ ^41^ ^]^ Notably, sensors using *Epipremnum aureum* leaf patterns exhibit a higher sensitivity of 110 kPa^−1^ but again across a narrow linear range of 0–0.2 kPa.^[^ ^44^ ^]^ When these HS‐I are replicated through laser techniques, eliminating the biotemplates, they have shown sensitivity of 11.06 kPa^−1^ across a broad range 0.6–10 kPa^[^ ^50^ ^]^ and sensitivity of 4.48 kPa^−1^ with an even broader range of 0–22 kPa.^[^ ^55^ ^]^ This suggests that the fabrication method and actual design of HS‐I sensors matter significantly. Sensors with microdome microstructures fabricated with irregular sizes (HS‐II types of microstructures) can exhibit very high sensitivity up to 124 kPa^−1^.^[^ ^17^ ^]^ As a comparison, sensors with conventional microdomes have a sensitivity of only 1.82 kPa^−1^.^[^ ^60^ ^]^ The HS‐II sensors have an overall excellent linearity range of 0–1700 kPa but a limited sensitivity of 0.065 kPa^−1^.^[^ ^36^ ^]^ Other conventional microstructures, such as micropyramids, and micropillars structured sensors can also show high sensitivity comparable to robust bioinspired microstructure, but their linearity is largely restricted in the low‐pressure regime.

Flat sensors exhibit limited performance, whereas microstructured especially bioinspired hierarchical designs can significantly enhance both sensitivity and linearity, which are critical for high‐performance pressure sensing applications. A flat sensor exhibited a sensitivity of 0.37 kPa^−1^, whereas the introduction of microstructures enhanced the sensitivity significantly to 4.48 kPa^−1^.^[^ ^55^ ^]^ Further emphasizing the advantages of bioinspired microstructuring, a study compared single‐level and hierarchical microstructures. The single‐level design demonstrated a sensitivity of 0.45 kPa^−1^ in the 0–10 kPa range, while the two‐level hierarchical structure (HS‐I) achieved a much higher sensitivity of 11.06 kPa^−1^ in the 0.6–10 kPa range.^[^ ^50^ ^]^ Hierarchical structuring (HS‐II) using a gradient microdome structure extended the sensor's linearity range and sensitivity. The gradient design provided a sensitivity of 0.065 kPa^−1^ over a broad 0–1700 kPa range, compared to 0.069 kPa^−1^ from uniform microdomes limited to a 0–100 kPa range.^[^ ^36^ ^]^

Bioinspired structures such as interlocked spinosum, cilia, and other hierarchical designs have been developed to enhance sensor performance beyond that of conventional microstructured and non‐microstructured sensors. While biotemplating offers a sustainable and facile fabrication route, it lacks scalability and reproducibility. In contrast, advanced techniques like lithography and laser‐based methods provide greater precision and scalability. Skin‐inspired interlocked spinosum structures fabricated using laser techniques are the most performant, with the requirement of several parameter optimizations. Also, fabrication strategies play an important role in fabricating robust microstructures. Photolithography and templating approaches provide robust, repeatable microstructures, showing excellent durability. Laser and magnetic‐field‐based techniques offer more scalable methods to fabricate microstructured surface with limited durability. Ultimately, application‐specific optimization is necessary to ensure reliable and stable performance in robotic materials.

In nature, sensing and actuation are inherently integrated. Bioinspired microstructuring strategies are interesting not only to mimic the sensing of organisms, but also their actuation, as organisms continuously react to their environments. Having explored how microstructures enhance sensing performance, it is equally important to examine how similar bioinspired structural principles can be applied to actuation. The next section, therefore focuses on how bioinspired microstructures can elevate the performance of actuators.

## 3 Actuators With Bioinspired Microstructures

Bioinspired microstructured actuators are motion‐generating devices that emulate the complex, efficient, and multifunctional mechanisms found in natural materials via microstructuring. Unlike conventional actuators that rely on multiple parts or components joined together, which limites motion complexity, bioinspired actuators utilise complex hierarchical microstructures such as the helicoidal fiber arrangements found in muscles or the stiffness gradient found in pinecone scales to achieve high degrees of complex, reversible, and energy‐efficient deformations. In this section, we discuss bioinspired microstructured designs for actuators, their fabrication methods, and compare their performance to comment on the best‐suited designs for targeted applications.

### 3.1 Bioinspired Microstructures for Actuators and Their Fabrication

To induce motion inside a single material without employing joints, non‐uniform stress should be patterned inside the material. Thus, the deliberate spatial variation of internal stress or strain fields within a material will depend on the material's fabrication process, its chemistry, its structure, and the stimulating mechanisms, thus resulting in controlled, asymmetric deformation. Typically, bioinspired microstructures create local anisotropy, inducing local shrinkage or expansion upon a stimulus. The most well‐known examples are the pinecones that open and close with humidity due to a bilayer morphology with perpendicular directions of shrinkage in each layer.^[^ ^61^ ^]^ Similar mechanisms are found across various other plants to achieve bending, twisting, or combinations of these motions.^[^ ^62^ ^]^ For bioinspired microstructured actuators, a large range of microstructures have been explored, which are reviewed. Since there is a variety of actuators with different microstructures that exhibit varying actuation behaviour, we have broadly distinguished them based on arrangement and complexity. These are unidirectional, including nacre‐like and other layered microstructures; fibrillar, which typically consists of fibrous arrangement, helicoidal and tubular microstructures; complex microstructures that include bilayer, gradient, janus, and other combinations of microstructures such as helicoidal and tubular; lastly, there are some other microstructures such as mushroom‐shaped, turing, etc., all of which we have discussed in details in subsequent sections.

**Figure 5:**
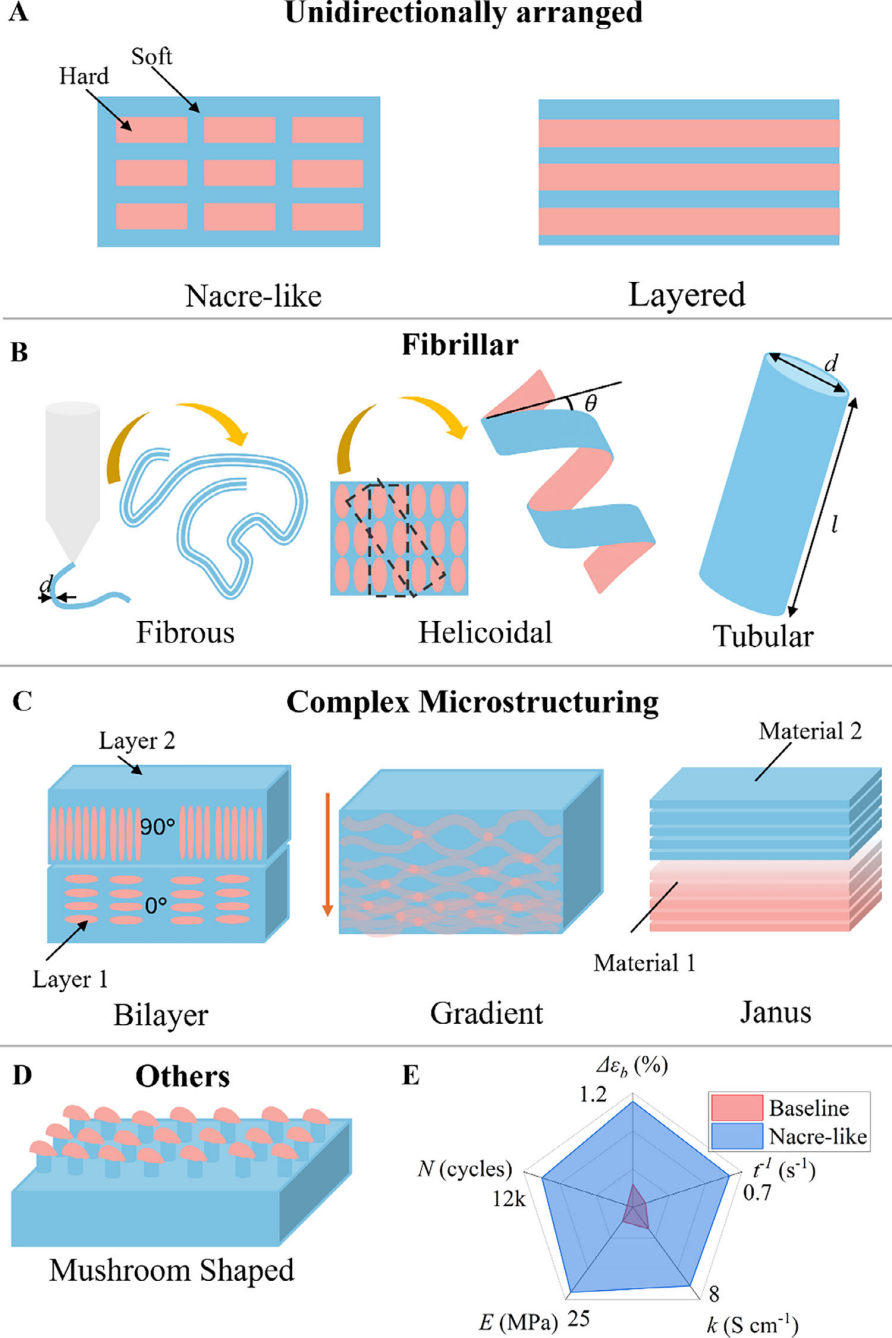
Schematics showing common bioinspired microstructures in four categorised microstructures. **A)** Unidirectional category consisting of sheet‐like layered structures, such as nacre‐like and other layered microstructures **B)** Fibrillar containing fibrous, helicoidal, and tubular microstructures, Reproduced with permissions from ref.[64] Copyright 2024, John Wiley and Sons. *d* is diameter, *l* is length parameter, *θ* is helix angle in Fibrillar Structures. **C)** Complex microstructures containing bilayered and other combinations of microstructures, and **D)** Other microstructures containing mushroom‐shaped and other microstructures. **E)** Radar plot showing overall improvement in properties due to bioinspired microstructuring, where *Δε*: bending strain difference, *t*: response time to peak actuation, *k*: electronic surface conductivity, *E*: Elastic modulus and *N*: Durability. The data used to plot it is taken from ref.[63]

One of the important points to be taken note of is that typical bioinspired actuators provide better mechanical, functional, and enhanced actuation properties compared to the baseline materials (materials without bioinspired microstructures). In order to provide an understanding to the readers, nacre‐like microstructuring can be compared to its reference baseline material under identical geometry, voltage (± 3 V), frequency (0.1 Hz) and ambient conditions as the nacre‐like carbon‐nanomesh (CNM) device (**Figure** **5**E).^[^ ^63^ ^]^ Indeed, it is well evidenced that the bioinspired microstructuring enhances the bending strain difference, *Δε* by 4.6 times, while being 35% faster with a durability of at least 15 times more than the baseline material. Since this shows that the bioinspired microstructure indeed plays a key role in enhancing the performance of actuators, therefore, we restrict our discussion to bioinspired microstructures in the subsequent sections.

**Figure 6:**
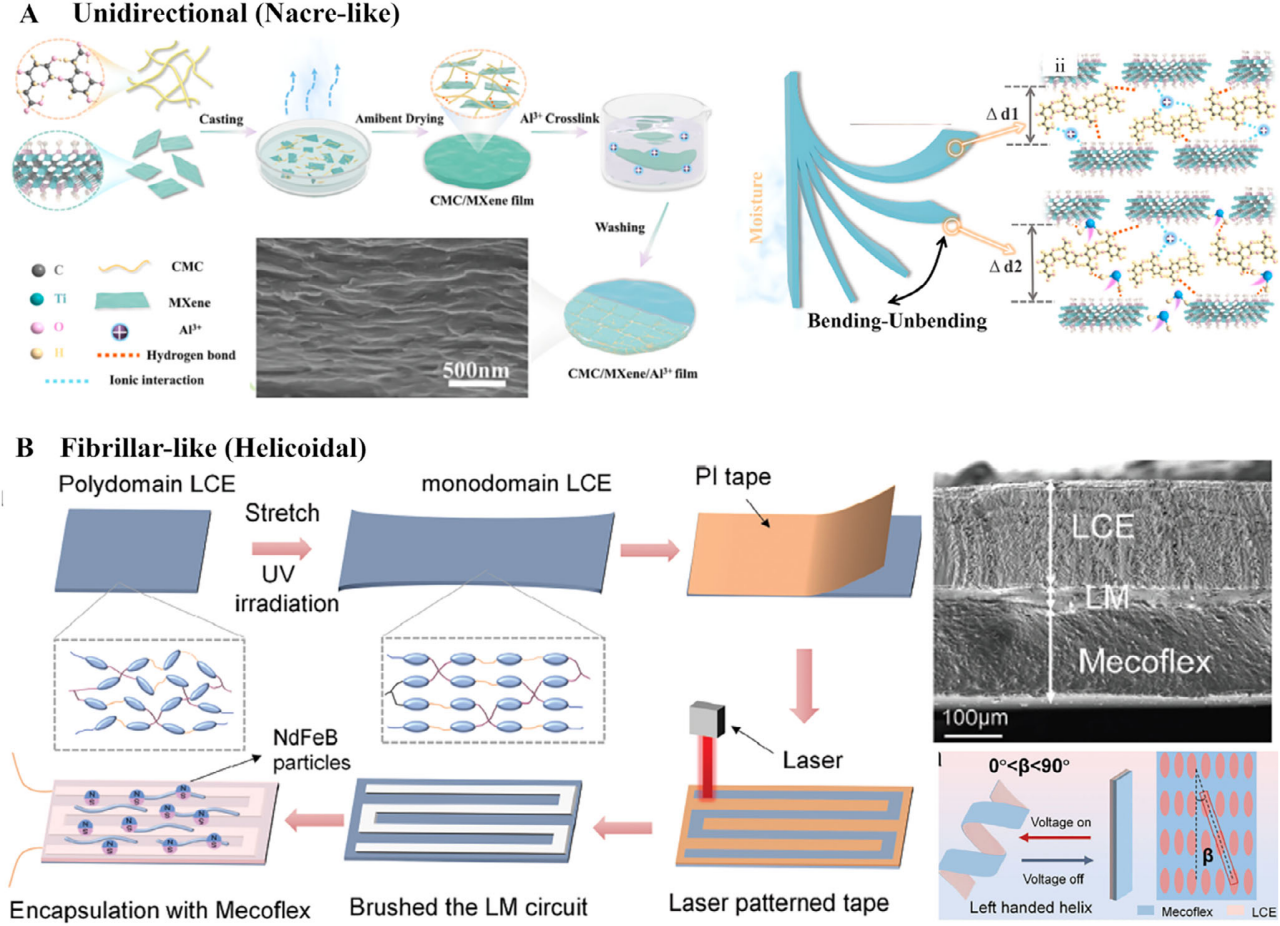
Fabrication techniques of nacre‐like and fibrillar‐like (Helicoidal) microstructured actuators with their actuation principle. **A)** Schematics of casting process and electron micrograph of the created nacre‐like microstructure (left), with the action motion on the right; CMC: carboxymethyl cellulose. Reproduced with permissions from ref.[65] Copyright 2023, Elsevier. **B)** Schematics of the fabrication process coating, casting and laser cutting and curing to create helicoidal microstructured actuators; LCE: liquid crystal elastomers; LM: Liquid Metal. Reproduced with permissions from ref.[64] Copyright 2024, John Wiley and Sons.

**Figure 7:**
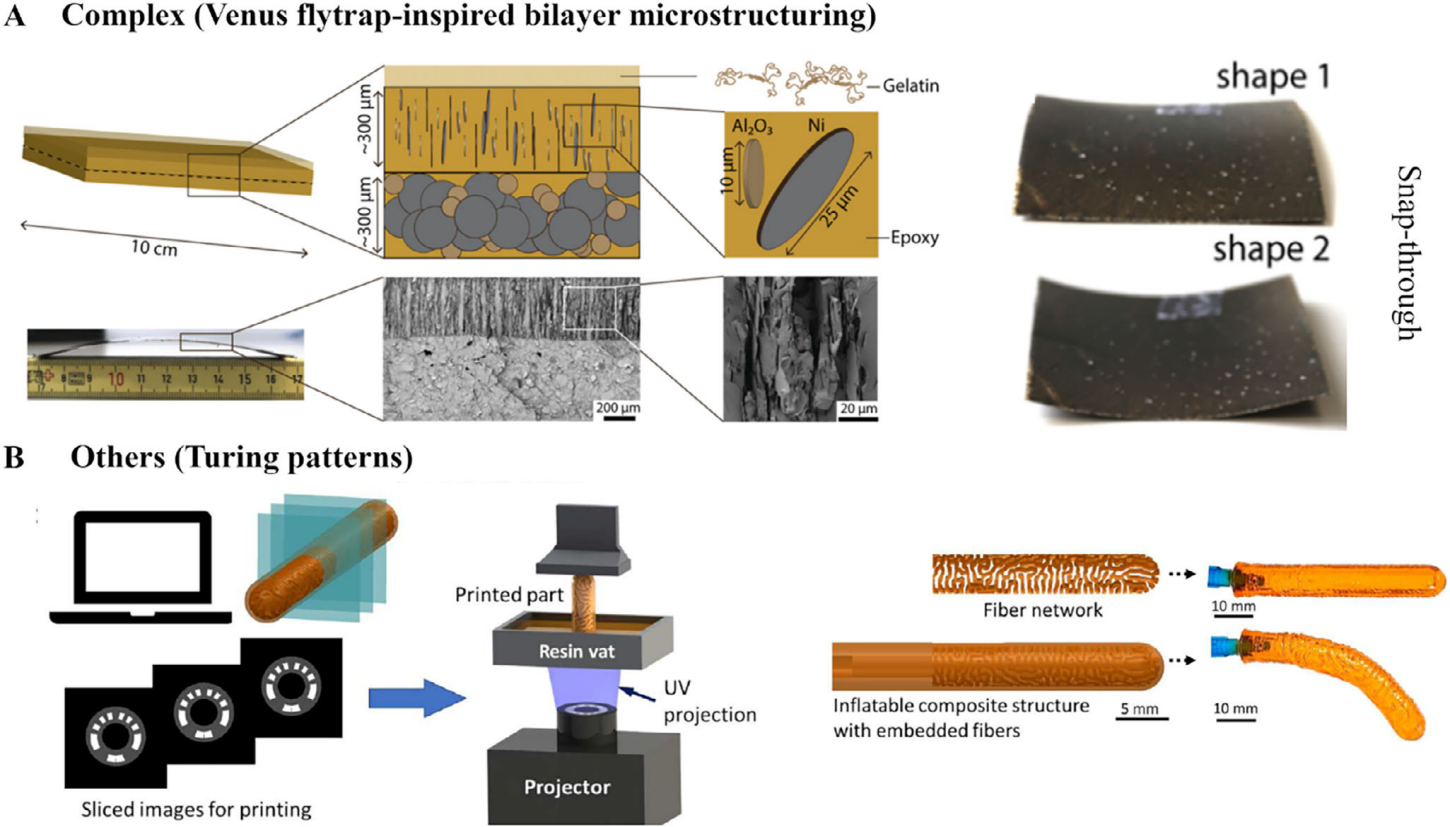
Fabrication techniques of complex arrangement microstructure and other microstructures for actuators. **A)** Schematics of the multilayered casting process inspired by Venus flytrap (left) and the actuation process by snapping (right). Reproduced with permissions from ref.[83] Copyrights 2022, IOP Publishing. **B)** Schematics of the digital light‐based 3D printing technique, processing to obtain Turing patterned microstructure. Reproduced under terms of the CC‐BY license.^[^ ^100^ ^]^ Copyrights 2023, Masato Tanaka, S. Macrae Montgomery, Liang Yue, Yaochi Wei, et al., published by AAAS.

The range of motion of those actuators, their motion complexity, their stimulus mechanism, etc., is detailed for each category along with their fabrication methods (**Figures** **6**, **7**), and each of them is described in detail to elucidate their role.

#### 3.1.1 Unidirectional

Several studies have adopted nacre‐like “brick‐and‐mortar” microstructures to marry mechanical resilience with programmable responsiveness (Figure 5A; Table s3, Supporting Information).^[^ ^63^, ^65^, ^66^, ^67^ ^]^ This bioinspired design achieves its functionality through mechanics dominated by shear transfer and crack deflection between stiff platelets and the soft matrix.

Nacre‐like microstructures, modelled after the nacreous layer found in seashells, consist of stiff nanosheets such as graphene oxide (GO), MXene, carbon nanomeshes embedded in soft polymer matrices and aligned in a layered fashion. Such microstructures can be processed through techniques like evaporation‐induced self‐assembly, casting, and ionic crosslinking (Figure 6A, left and centre), and digital slicing/cutting strategies. One method introduced a cutting‐and‐stacking approach to form nacre‐like 3D curved composites with staggered ply cuts that eliminate weak overlap zones, achieving up to 69 % higher compressive strength in 3D actuator shells.^[^ ^66^ ^]^ Nacre‐like microstructuring imparts high strength, flexibility, and multi‐stimulus responsiveness. For example, a CMC/MXene/Al^3^⁺ actuator film bends at 92 s^−1^ under humidity gradients, retains a wet tensile strength of 154 MPa at 97 % RH, and survives >1500 cycles (Figure 6A, right).^[^ ^63^ ^]^ Likewise, integrating nacre‐derived carbon nanomeshes into ionic‐polymer actuators enhanced ion transport and long‐term actuation under low voltages.^[^ ^63^ ^]^ Overall, nacre‐like microstructuring provides a structurally efficient, multifunctional platform for soft robotics, energy harvesters, and smart adaptive materials.

On the other hand, layered lamellar microstructures have a sheet‐like arrangement, in contrast to the interpenetrating “mortar” phase of nacre, thereby enabling stronger directional actuation. Such lamellar microstructures are either formed by self‐assembly in response to changes in environmental conditions or external forces. Polymers like sodium acrylate‐based they typically undergo water‐assisted microphase separation to form nanoscale lamellar domains.^[^ ^68^ ^]^ With further process regulation by hydrophilic/hydrophobic balance, the resulting lamellar films can expand/contract reversibly with humidity, producing macroscopic bending and making them ideal for moisture‐sensitive sensors and actuators. Similarly, a gecko‐inspired magnetic lamellar array, when exposed to magnetic field shows a three‐fold difference in transport capacity between forward and reverse motion by exploiting slanted geometry and elastic energy storage.^[^ ^69^ ^]^ The anisotropic geometry causes a three times difference in transport capacity between forward and reverse directions, making them suitable for capsule delivery and solid object manipulation in curved environments. In 3D‐printed bismuth‐sodium‐titanate (BNT) piezoceramic actuators, lamellar grain–matrix interfaces raise the piezoelectric strain to 0.26 % and deliver a 15 N blocked force.^[^ ^70^ ^]^ The lamellar microstructure facilitates efficient field‐induced bending by enhancing dielectric continuity, enabling curved, compact actuator designs with improved electromechanical coupling. Furthermore, shear‐assisted polymerisation has been used to create unidirectional lamellae in a hydrogel that stretch 3200% and swell anisotropically, perpendicular to the layers of 210% versus 35% in parallel.^[^ ^71^ ^]^ The ordered microstructure not only guides mechanical reinforcement but also facilitates photo‐thermal actuation and ionic conductivity through the intercalated polypyrrole (PPy) and lithium chloride (LiCl) domains. The materials demonstrate somatosensory actuation, lifting loads ≈248 times its dry weight, and operate durably across thermal and strain cycling. The lamellar microstructures, therefore, combine structural anisotropy with stimuli responsiveness, bridging moisture sensors, directional transport, and high‐strain actuation to address critical needs in soft robotics, wearable electronics, and human‐interactive devices.

#### 3.1.2 Fibrillar Microstructures

Fiber‐based systems translate the hierarchical, high‐strain mechanics of muscle into scalable actuators. Fibrous microstructures typically contain cylindrical microstructured fibers with a certain diameter and length (Figure 5B, left). Thermal drawing of bimorph fibers (High Density Polythene / cyclic‐olefin‐copolymer elastomer) achieves >1000% strain tolerance and 50% contraction at ΔT = 14 °C, with power‐to‐mass ratios of 90 W kg^−1^ surpassing human skeletal muscle by an order of magnitude.^[^ ^72^ ^]^ These fibers can be drawn into kilometer long continuous threads with diameters spanning µm to mm and then cold‐drawn to create tendril‐like springs. Incorporating Ag‐nanowire meshes confers proprioceptive sensing for >10⁵ cycles while promoting multifunctionality, such as simultaneous actuation and intrinsic sensing. Electrospun microribbons coated with Au/polypyrrole (PPy) curl reversibly under ± 0.6 V and self‐sense loads up to 21 mg.^[^ ^73^ ^]^ A pennate‐inspired fiber‐bundle actuator (HimiSK) with embedded McKibben muscles has been used to reproduce force‐length/velocity curves and load‐responsive gearing without external sensors.^[^ ^74^ ^]^ As a whole, the fiber‐based microstructures thus underpin muscle‐like, sensor‐free soft‐robotic limbs and high‐power artificial tendons that are crucial for producing scalable, adaptive, and biologically accurate actuator systems.

Helicoidal arrangements impose kinematics constraints and thus convert fiber twist into torsion, lift, and torque by taking cues from plant tendrils and insect setae; therefore, versatile motions like bending, twisting, coiling, and length changes can be achieved.^[^ ^75^ ^]^ Typically, they contain predominantly fibers arranged in a helicoidal or helix fashion with a defined helix angle (Figure 5B, centre). The mechanics arise from fiber‐reinforced anisotropy. Biomimetic helical fiber actuators have been designed using several ranges of elastic materials like liquid crystal elastomers (LCE), shape memory materials, and fibers like carbon, glass, and even ceramics. Their fabrication process is unique based on material choice. Using methods like wet/dry spinning and twisting, materials such as LCEs and carboxymethyl cellulose (CMC) can be shaped into yarns or multi‐strand fibers with tightly wound helical geometry (Figure 6B).^[^ ^64^ ^]^ Facile twisting and two‐step crosslinking helped to integrate LCE fiber as the actuation phase with bacterial cellulose (BC) fibers to produce stiff actuators exhibiting mechanical strength up to 43.9 MPa and work capacity of 304.1 J kg^−1^.^[^ ^76^ ^]^ It is interesting that in such actuators, yarn twisting angle (β) is an important consideration for excellent mechanical properties, and it increases with the number of twists. These structures reduce chain slippage, resist creep, and support multifunctional responses, especially when combined with programmable magnetism or light‐thermal conversion layers for multimodal actuation. Apart from the number of twists, the number of strands also enhances actuators with high strength, toughness, and a large load. Multi‐strand CMC fibers with hierarchical helicoidal morphology under moisture stimulation allow actuation equivalent to lifting 20000 times their own weight.^[^ ^77^ ^]^ Furthermore, advanced helicoidal actuator designs can be created via 4D printing of bilayer tubular metacomposites, allowing for the tuning of twist angle, layer ratio, and strand number to achieve shape change, torque, and blocked force outputs that mimic those of biological fibers.^[^ ^78^ ^]^ These works highlight helicoidal architecture as a structurally efficient strategy to achieve programmable, reversible, and multifunctional deformation modes with promising mechanical performance that could enable applications from bioinspired robotics to adaptive wearables.^[^ ^77^, ^78^ ^]^

The tubular architectures that are predominantly hollow tube‐like structures offer a unique platform to encode complex, muscle‐like deformation modes within a continuous and compact body (Figure 5B, right). Researchers developed hydrothermally responsive tubular soft actuators by embedding twisted cotton yarn into a hydrophilic polyurethane tube, mimicking the muscle arrangements of an elephant trunk.^[^ ^79^ ^]^ Upon exposure to water or temperature changes, these actuators undergo programmable deformation, achieving linear elongation of ≈40%, reversible contraction of ≈10%, and multi‐axis motions, including bending and rotation, through a strategic outer layer coating. Their fabrication is facile, which is achieved via dipping, coiling, and patterning. Similar to how mammals contract muscles in different body parts, electrically actuated LCE‐based tubular actuators can be designed with embedded serpentine heating wires that are independently controllable. The actuators exhibit electrically controlled bending and uniform contraction up to 41% with programmable, multimodal outputs.^[^ ^80^ ^]^ Their response is rapid, reversible, and durable over 100 cycles, achieving actuation stresses ≈0.35 MPa and work densities ≈150 kJ m^−^^3^ comparable to mammalian skeletal muscle. Overall, the hollow geometry eases wiring, fluid delivery, and on‐board power, enabling untethered crawling, grasping, and pumping suitable for the development of intelligent, adaptive soft robotic systems.

#### 3.1.3 Complex Microstructuring

The complex microstructures predominantly combine or arrange two or more microstructural arrangements (Figure 5C). One of the complex microstructures is the bilayered microstructuring, where one layer having a certain degree of microstructural organisation is organised in a deliberate manner over another to benefit from local microstructuring (Figure 5C, left). Thus, this exhibits programmable and bioinspired shape transformations by embedding spatially controlled reinforcement architectures within responsive matrices.^[^ ^81^ ^]^ Unlike homogeneous materials, these microstructured composites leverage anisotropic fillers such as magnetically aligned alumina platelets that are initially magnetized, to locally restrict swelling, shrinkage, or thermal expansion. The behaviour of plant structures (e.g., pinecones, seedpods) has been replicated by aligning iron oxide‐coated microplatelets under low magnetic fields, forming bilayers that bend or twist upon hydration.^[^ ^82^ ^]^ The resulting anisotropy‐induced directional swelling strains up to 2.5 times greater than the perpendicular direction of the reinforcement, with shape change governed by bilayer mechanics. Moreover, using Timoshenko‐based model, the curvature depends on the modulus and thickness ratio of the layers, enabling precise shape prediction and actuation control. Building on this, another work integrated bistability into [0°/90°] bilayer epoxy composites topped with gelatine hydrogels, enabling both fast snap‐through morphing ≈50 ms and slow hydration‐driven actuation in hours using hierarchical microstructuring (Figure 7A).^[^ ^83^ ^]^ The process used simple magnetically assisted slip casting (MASC) to achieve the hierarchical assembly.^[^ ^84^ ^]^ In the hierarchical architecture, the pre‐stress is induced by curing above the epoxy glass transition temperature ≈102 °C that stores internal strains to trigger bistability, while the hydrogel layer allows reversible deformation. Interestingly, these systems respond to mechanical, thermal, magnetic, and hydration stimuli and exhibit multi‐zonal, site‐specific deformation through tailored reinforcement orientation, with further validation that the complex geometries, such as in Venus flytrap‐inspired morphologies, could be achieved by tuning microstructural layouts through finite element modelling. This demonstrates the critical role of complex microstructuring in enabling multi‐temporal, multifunctional actuator behaviour.

The gradient microstructures in actuators are arranged to perform a specific function, enabling site‐specific deformation and functionality (Figure 5C, center). Four strategies have emerged to enable gradients: i) cross‐link‐density gradients, ii) dual gradients that preload elastic energy, iii) alignment/anisotropy gradients, and iv) filler/composition gradients. The gradients can be introduced by UV photopatterning, electric/magnetic‐field induction, infiltration, wettability control, or grayscale additive manufacturing.^[^ ^85^ ^]^ Crosslink density gradients encoded via grayscale stereolithography in a hydrogel precursor enable reversible, site‐selective bending or twisting under uniform hydration stimuli, achieving curvatures greater than 1 cm^−1^ for more than 100 cycles. The complexity in gradient design was further enhanced using a dual‐gradient hydrogel combining polymer chain and crosslinking gradients, enabling elastic energy storage and rapid snapping motions in <1 s, mimicking the Venus flytrap mechanism.^[^ ^86^ ^]^ Interestingly, introducing an alignment gradient in nanofibers results in millisecond‐scale, multimodal shape transformations across dimensions from 0D to 3D, showcasing the versatility of structural anisotropy.^[^ ^87^ ^]^ Gradient architecture significantly enhances actuator performance by offering faster response times, multifunctionality, and higher mechanical robustness. This has been well demonstrated by hydrogels with a gradient in nanosilver flake concentration, achieving ultrafast thermal actuation of 52.3^°^/s and high conductivity >1231 S m^−1^ that is useful for both soft grippers and sensors.^[^ ^88^ ^]^ Furthermore, introducing a nacre‐like gradient design of MXene fillers in an epoxidized natural rubber (ENR) elastomer yielded a high tensile strength ≈ 25.03 MPa with a rapid photothermal response of bending to 92° in 2.4 s and a self‐healing efficiency of 91.7%.^[^ ^89^ ^]^ Furthermore, with a pore‐size/swelling gradient in a bilayer hydrogel, full 360° curls in 9 s can be achieved and are capable of morphing into flower, leaf, and butterfly‐like geometries.^[^ ^90^ ^]^ Across all these works, spatial gradients consistently shorten response times (ms‐s), integrate multiple functions (actuation, sensing, and self‐healing) and raise mechanical durability.

Janus microstructures extend graded designs by stacking layers with starkly different physicochemical properties (Figure 5C, right) and thus rely on bilayer mechanics. Two recurring design archetypes emerge: i) conductive moisture‐sensitive bilayers, and ii) photothermal‐mechanical bilayers that also offer structural colour. A flexible conductive hygroscopic Janus bilayer actuator fabricated by vacuum‐filtering conductive MXene onto hygroscopic cellulose‐nanofibre (CNF) paper couples the CNF's moisture swelling with MXene's high electro‐/photothermal conversion.^[^ ^91^ ^]^ Coupled with a printed MXene/polyethylene strain sensor, the actuator achieved multi‐responsive bending under light, electric, and humidity stimuli while simultaneously monitoring its own deformation with a linear resistance‐strain correlation. The bending curvatures achieved were significant, reaching a curvature of ≈ 4.39 cm^−1^ electrically, and ≈ 1.81 cm^−1^ from humidity shifts, along with a reliable cyclic stability over 50 cycles. Furthermore, embedding reduced graphene oxide (rGO) into the bottom of a cholesteric liquid‐crystal‐elastomer (CLCE) sheet yields a Janus‐like gradient during anisotropic deswelling.^[^ ^92^ ^]^ The rGO rich bottom layer provided mechanical anisotropy and photothermal responsiveness, while the CLCE rich top layer maintained vivid structural colour. Upon Near Infrared (NIR) irradiation, the resulting single‐layer film underwent reversible 3D deformation, simulating natural motions like blooming petals, twisting tendrils, and contracting webs. The Janus‐induced actuation was not only mechanically stiff but also optically dynamic, enabling simultaneous motion and colour response. Together, these works highlight that Janus architecturing enables simultaneous actuation, sensing, and visual feedback in a single laminate, eliminating the need for multi‐part assemblies and advancing untethered soft robots and smart wearables.

The combined architectures in actuators to integrate multiple structural motifs such as helicoidal, tubular, gradient, and nacre‐like designs exploit synergistic effects in deformation control, mechanical strength, and multi‐functionality that are hard to achieve in a single motif. Bio‐inspired helical‐artificial‐fibrous‐muscle structured tubular soft actuators embed wound fibres inside an inflatable shell; by tuning the winding angle *θ*, a single body accesses 11 morphing modes, bending, twisting, phototropic or photophobic turning, volumetric pumping, while locally switching Poisson's ratio (*ν* = 0 or *ν* < 0), enabling unique volumetric transformations.^[^ ^93^ ^]^ Helix‐tubular composites made from braided ultra‐high molecular weight polyethylene over knitted polypropylene cores replicate the triphasic stress‐strain signature of ligaments and withstand >1500 fatigue cycles with strain‐dependent conformation switching, thus delivering tendon‐like function.^[^ ^94^ ^]^ With further addition of gradient distributions to the multi‐architecture systems, performance is enhanced with programmable response speed, self‐healing, and higher load capacity. Together, these works demonstrate that combining structural principles such as helicoidal, tubular, and gradient distribution yields actuators with programmable, robust, and multifunctional responses that are promising for next‐generation soft robotics.

#### 3.1.4 Others

Mushroom‐shaped architectures, typically in the shape of a mushroom in actuators, provide a multifunctional design strategy (Figure 5D) by combining adhesive control and active deformation, inspired by gecko toe pads.^[^ ^95^ ^]^ A light‐driven untethered actuator with integrated mushroom‐tipped micropillar arrays has been demonstrated on a photothermally responsive PDMS/MWCNT (polydimethylsiloxane/multiwalled carbon nanotube) trilayer structure.^[^ ^96^ ^]^ Fabricated via soft lithography and ink‐imprinting, these micropillars offer switchable dry adhesion that is modulated by light‐controlled deformation. Under NIR irradiation, MWCNTs convert light into heat, causing PDMS expansion and bending that enables both locomotion i.e, rolling or crawling; and object manipulation via adhesion‐on/adhesion‐off switching. The mushroom geometry enhances van der Waals contact, enabling adhesion across curved or irregular surfaces, while the photothermal actuation allows untethered operation without mechanical components. This microstructure supports applications in grasping, targeted delivery, and terrain‐adaptive microrobots, providing a bioinspired route toward light‐controlled adhesion actuation coupling in soft robotics.

The Turing patterns arising from reaction‐diffusion systems are basically self‐organised patterns that offer a powerful bioinspired framework for encoding complex material anisotropy into actuator microstructures.^[^ ^97^ ^]^ It was initially formulated to explain morphogenesis,^[^ ^98^ ^]^ however, these patterns can now be computationally generated and harnessed to map optimized anisotropic material orientations into manufacturable, isotropic architectures.^[^ ^99^ ^]^ Tanaka et al. demonstrated a strategy to convert continuous orientation fields obtained via gradient‐based optimization for target shape morphing into binary Turing patterns using anisotropic reaction‐diffusion equations.^[^ ^100^ ^]^ These patterns, representing alternating soft and stiff regions, were then directly fabricated using grayscale digital light processing, which modulates local stiffness via light intensity in a single resin vat (Figure 7B). The result is a high‐resolution composite that mimics the mechanical function of the original anisotropic design while remaining fully compatible with voxel‐based additive manufacturing. The Turing‐patterned actuators exhibit programmable, directional deformation driven by internal heterogeneity, enabling reversible shape transformations such as bending, twisting, and 3D morphing in soft robotic elements.^[^ ^100^ ^]^ The binary material layout derived from the reaction‐diffusion system effectively preserves the mechanical anisotropy necessary for actuation despite using isotropic base materials. Finite element simulations validated that deformation behaviour closely matches the original orientation‐optimized designs, with tunable fiber angles encoded into the Turing stripe geometry. This approach bridges the gap between computational inverse design and fabrication feasibility, offering a robust platform for creating scalable, bioinspired actuators. The use of natural patterning principles for structural encoding thus represents a new paradigm in actuator design, where complex, stimuli‐responsive behaviour can emerge from simple fabrication constraints.

The fabrication methods, such as casting, deposition, 3D printing, laser cutting, hierarchical assembly via sequential casting, etc. have been used to create a variety of bioinspired microstructures. In the next section, we analyse their performance metrics to understand their potential applications and limitations.

### 3.2 Performance of Actuators with Bioinspired Microstructures

**Figure 8:**
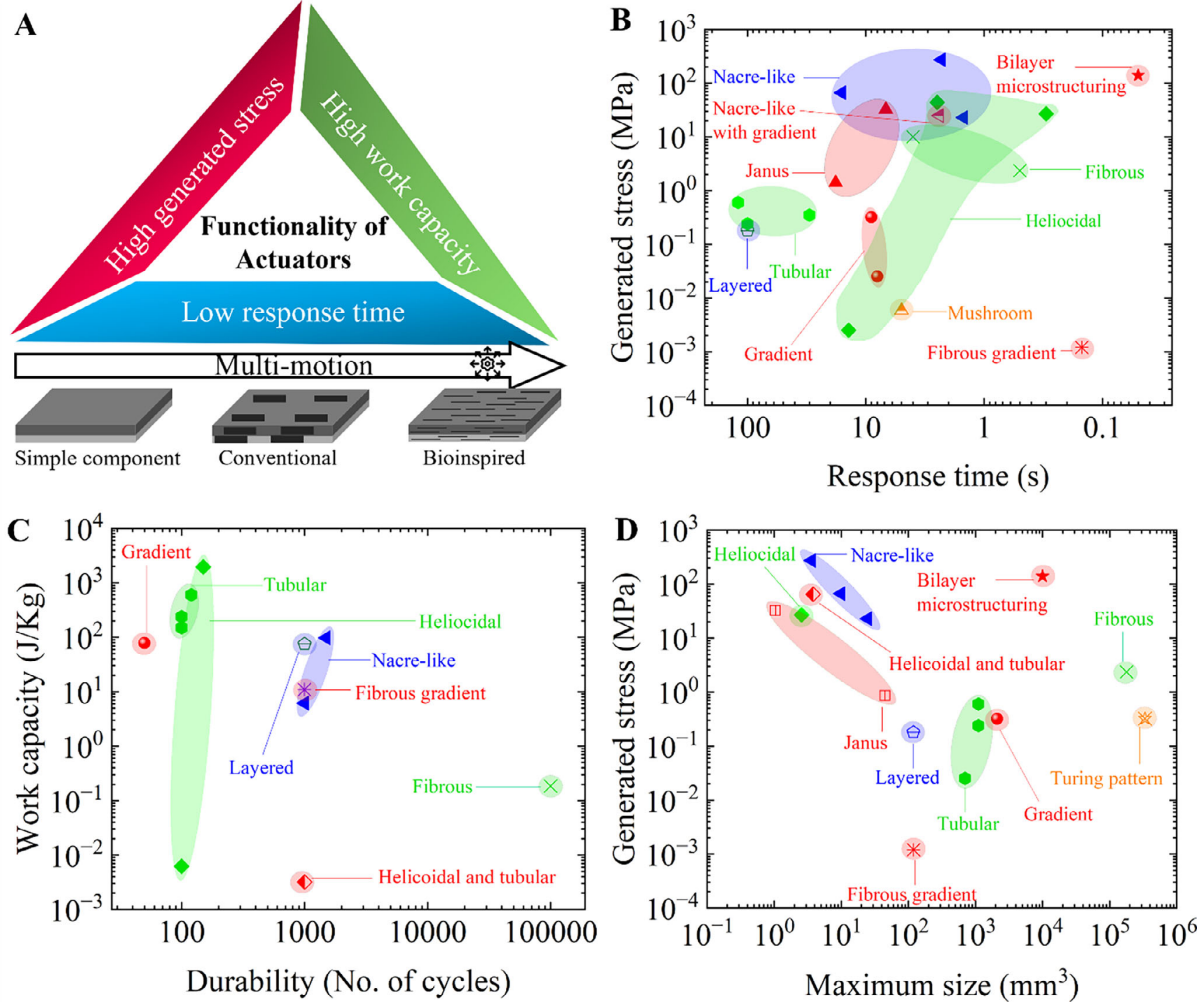
**A)** Key performance metrics that determine the functionality of the actuators. Comparison of the bioinspired microstructured actuators in terms of **B)** Generated stress or Output stress versus response time. **C)** Work capacity versus Durability and **D)** Generated stress versus maximum build size in volume of the actuators. All the raw data are available in Table s2 (Supporting Information). The colour codes stand for blue: unidirectional, green: fibrillar, red: complex, and orange: others.

Actuators with bioinspired microstructures integrate such microstructures in order to mimic the movements and dynamics of natural organisms. Their performances of such state‐of‐the‐art actuators are collected and compared in performance maps (**Figure** **8**; see Table S2, Supporting Information for the aggregated data) by distinguishing each category with unidirectional represented by blue, fibrillar by green, complex by red, and other microstructures by orange (Figure 8).

The performance of bioinspired actuators can be evaluated by three key metrics: stress generated, response time, and work capacity (Figure 8A).^[^ ^75^, ^101^ ^]^ The generated stress represents how much internal force the actuator material can exert on its surroundings, while the response time determines how fast or slow the actuator can execute the motion. The work capacity determines the output work efficiency, which is useful for determining the movement per unit mass. Additionally, durability is a crucial factor that ensures consistent performance over time for precise movements, preventing unexpected failures and disruptions. These metrics are particularly important where the actuator devices with diverse technologies are used for applications ranging from biomedical devices to aerospace. With these metrics, researchers and engineers can objectively compare different actuators and identify the most suitable options for specific applications, ensuring that the chosen device meets the functional requirements of the target use case.

The generated stress versus response time is compared for various bioinspired microstructured actuators (Figure 8B; see Table S2, Supporting Information for values). The actuators with nacre‐like microstructures generate the highest stresses varying from 23 to 273.6 MPa, followed by actuators with complex microstructuring inspired by the Venus flytrap, then the actuators with janus and nacre‐like with gradient microstructures, with the actuators generating the least stress being those with fibrous gradients. The higher stress in the predominant nacre‐like microstructure is due to the high packing fraction of reinforcing particles.^[^ ^63^, ^65^, ^66^, ^67^ ^]^ The variations in the generated stresses are due to different material compositions. In turn, the actuators with fibrous graded microstructures generate the lowest stresses due to the low concentration of fibers on one side.^[^ ^87^ ^]^ However, the actuators with fibrous graded microstructure have a fast response time of 0.15 s, and the Venus flytrap‐inspired microstructure has the fastest response time of 0.05 s.^[^ ^83^ ^]^ The rapid response is predominantly attributed to the bistability of the bilayer structure with [0/90] architecture, which stores pre‐stresses during fabrication and enables the system to snap rapidly between two stable shapes. This behaviour imitates the fast closing of the Venus flytrap upon touch.^[^ ^83^ ^]^ Nevertheless, the observed actuation response decreases in general with the increased generated stress. Thus, actuators with nacre‐like, fibrous, layered, combined microstructures, helicoidal, tubular, etc., show actuation time response between 1 and 100 s. Interestingly, the response time is not only influenced by the microstructural design but also by the actuation mechanism, material properties, scale, operating environment, and the type of stimulus applied. For example, the actuators driven by magnetic or electrostatic fields typically exhibit the fastest response times, as these mechanisms rely on real‐time physical forces that can operate on the order of milliseconds.^[^ ^83^, ^87^ ^]^ Furthermore, the actuators with helicoidal microstructures offer a range of properties varying from 0.001 to 100 MPa generated stress and 0.3–10 s response time.^[^ ^64^, ^75^, ^76^, ^77^ ^]^ This is probably due to the complex interplay between their geometry and the materials used. The helicoidal topology enables a wide range of mechanical responses, including length contraction and elongation, twisting, bending, and coiling. Typically, these responses can be influenced by factors such as the bias angle of the fibers, the volume change of the matrix, and the inherent properties of the materials involved.

The work per unit mass versus durability for actuators with various bioinspired microstructures is compared. (Figure 8C). The actuators with helicoidal microstructures exhibit the highest work capacity of 1962 J Kg^−1^,^[^ ^77^ ^]^ while ones with helicoidal and tubular microstructures have the lowest ≈ 0.00318.^[^ ^94^ ^]^ The lowest work capacity may be due to limitations imposed by the materials and design that lead to weak inherent material properties, low strain mismatch and resulting deformation, low energy conversion efficiency due to more dissipated heat, thin film structure, and limited actuation strain. Interestingly, the work capacity continues to decrease following the trend from helicoidal, tubular, gradient, layered, nacre‐like, fibrous gradient, fibrous, to finally helix and tubular. The durability, however, in general, shows a trade‐off; the fibrous material being highly durable with 10^5^ cycles mainly due to their continuous or twisted fiber alignment, while the gradient being the least durable with 50 cycles. The durability of microstructured actuators is mostly influenced by the internal stress distribution. Nacre‐like microstructure can delay structural failure under cyclic loading. Thus, stress‐dissipating design considerations through bioinspiration and material choices are some important considerations for stable actuators during prolonged operation.

The generated stress of the actuators with the maximum possible build sizes of the actuators is compared for the various microstructures in order to assess their scalability and potential applications (Figure 8D). There is indeed a trade‐off between compactness and power. For example, industrial machinery and devices require high generated stress, whereas implantable devices prioritize compact size. Regardless, a versatile actuator would be beneficial to meet both ends. The actuators with nacre‐like microstructure exhibit the highest generated stress of 273.6 MPa, however, they have a limited size of 25 mm^3^. In contrast, the actuators with Turing patterns can be of a large size of 335714 mm^3^ but have a low generated stress of 0.3 MPa. All the other actuators appear to exhibit a general trade‐off between the generated stress and size. Interestingly, the Venus flytrap‐inspired bilayer actuators exhibit a good compromise of generated stress ≈ 140 MPa with a maximum possible actuator size of 10000 mm^3^.

To decide which actuator to choose, it is essential to align the performance metrics with the application's functional needs and constraints. For instance, if a device requires high surface complexity for advanced robotic grippers, actuators with programmable motion and high deformation curvature would be suitable, such as the Venus flytrap inspired bilayer complex microstructure. On the other hand, for applications in aerospace, where durability is critical in addition to high generated stress, actuators with fibrous or fibrous gradient microstructures may be more suitable. Thus, by systematically evaluating performance metrics and considering factors such as facile fabrication, fabrication rate, actuation decoupling, and under‐actuation, researchers can make informed decisions to optimize the design and functionality of actuating devices for their intended applications. Furthermore, such actuators can be coupled with microstructured sensing designs in order to perform computation at the materials level.

## 4 Bioinspired Microstructures for Creating Robotic Materials Coupling Sensing, Computation and Actuation

Bioinspired microstructures can be used to create sensors with high sensitivity and a broad linear range. At the same time, bioinspired microstructures can yield actuators with fast response, generating high stresses and a large work capacity. In robotic materials, sensing and actuation are coupled within the material to allow for computation. First, bioinspired microstructures, aided by hierarchical multi‐scale design, enable actuation that is inherently coupled with one or multiple stimuli. Second, complex hierarchical microstructures and ways of interpreting stimuli have been explored to embed computation at the material level.

### 4.1 Coupling Sensing with Actuation

**Figure 9:**
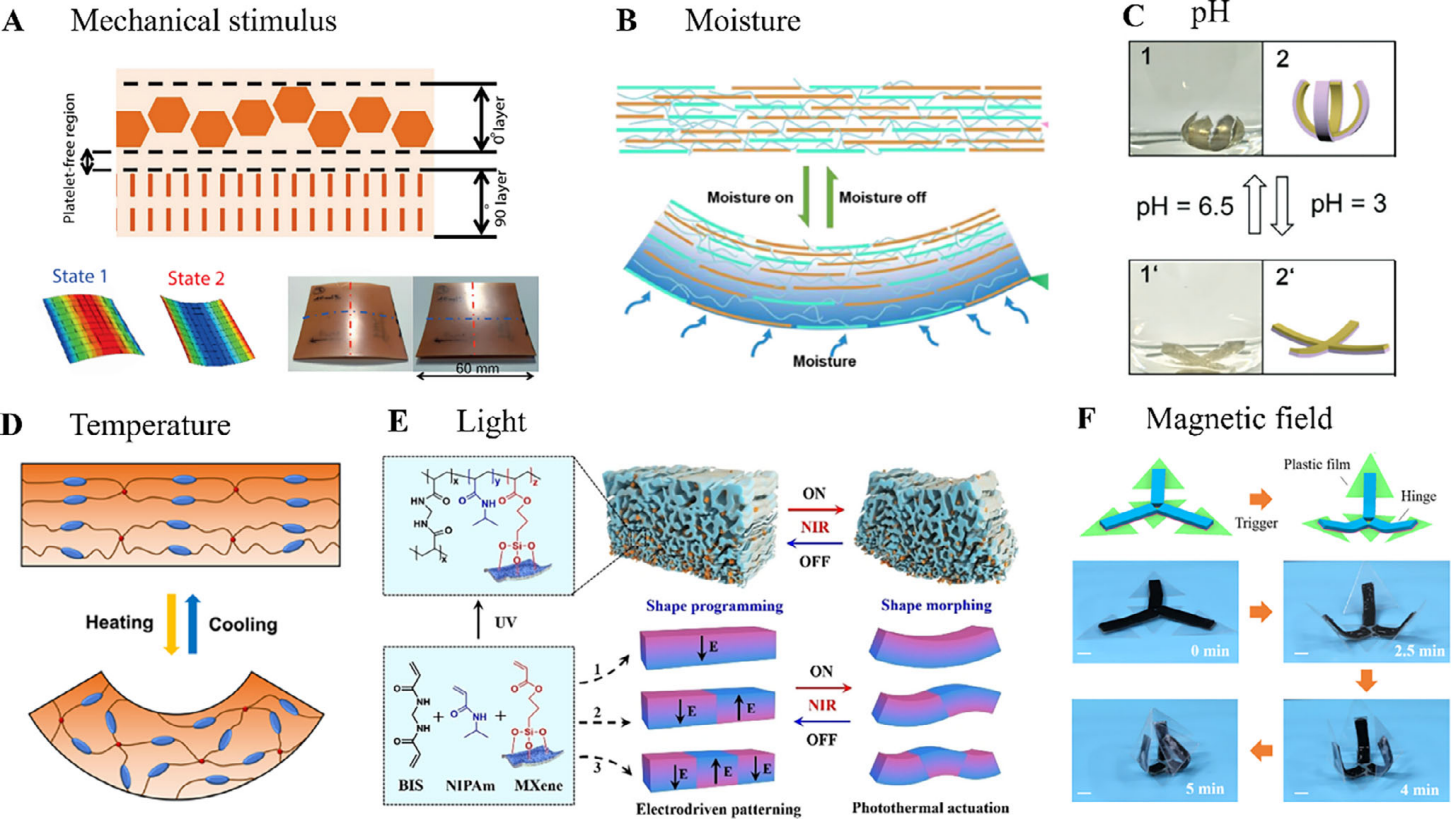
Working mechanisms of morphing and actuators with bioinspired microstructural design. **A)** Illustration of bilayer laminate made by tuning the filler orientation. Snap‐through as the mechanical stimulus triggers this bistable structure, showing two states. Reproduced with permission from ref.[102] Copyrights 2017, IOP Publishing, Ltd. **B)** Illustration of moisture triggering the morphing with gradient distribution of fillers. Reproduced with permissions from ref.[103] Copyrights 2022, Elsevier. **C)** Illustration of composites with ionizable groups in the hydrogel network, showing its morphing capability upon the tuning of solvent pH. Reproduced with permissions from ref.[104] Copyrights 2012, Royal Society of Chemistry **D)** Illustration of LCP with the mesogen orientation gradient design, controlled by 3D printing method to induce capability of reacting to temperature change and morph. Reproduced with permissions from ref.[105] Copyrights 2019, American Chemical Society **E)** Illustration of material with gradient porous microstructure sensitive to light. It transforms the NIR light energy to thermal energy by MXene fillers and induces heating to show the morphing. The microstructural design was induced by the application of UV light. Reproduced with permissions from ref.[106] Copyrights 2021, John Wiley and Sons**. F)** Illustration of composites capable of morphing upon the application of alternating magnetic field, which can heat the magnetic‐responsive particles in the hydrogel to change its shape. Reproduced with permissions from ref.[107] Copyrights 2019, American Chemical Society.

The previous section reviewed how bioinspired microstructured generated actuation by creating materials with local variations in stresses and strains. The deformation of the materials then occurred upon an external stimulus. Depending on the chemical nature of the materials employed, external stimulus can be of various nature, such as mechanical pressure, moisture, pH, temperature, light, and magnetic fields. Multiple external stimuli can also be combined or used selectively to trigger the deformation of the material. These various stimuli and examples are reviewed in the following. (**Figure** **9**).

Bistable composites inspired by the rapid shape‐switching of the Venus flytrap enable fast morphing under mechanical pressure.^[^ ^108^ ^]^ These laminates transition between stable states by storing strain energy until a threshold is reached, triggering a rapid release to a second stable state, which enables the actuator to act as 0/1 bit represented by two distinct states (Figure 9A).^[^ ^102^, ^109^ ^]^ The structure ceases motion after state‐switching, although damping oscillations may occur. A representative case demonstrated the use of a laminate structure with aligned fillers to induce bistability.^[^ ^102^ ^]^ With the temperature decrease during the curing of epoxy matrix, pre‐stress can be introduced as stored potential strain energy. High actuation stress can then be exhibited upon snap‐through within a short time, showing the fast‐morphing capability. The induction of intrinsic bistability relies on pre‐stress of each part during manufacturing, which can be achieved by methods like additive manufacturing with bioinspired microstructures to avoid traditional pre‐stretching during processing. The stress field for snapping‐through can come from pressure, moisture swelling, magnetic‐ or temperature‐field which broads the application range of bistable actuators. As such, matrices for building bistable actuators should be tough to absorb strain energy upon snap‐through rather than fracture. The threshold of snapping stress should also be aligned with the applications, which are determined by matrices, fibers, as well as geometry of laminates. Actuators should also not be too thick to show necessary curvature for pre‐stress as well as snapping‐through.

Diffusion‐based actuator employs water or solvent transmitting around fillers or through pores for swelling driven by chemical potentials, which exhibits significant curvature changes as morphing (Figure 9B). For instance, a gradient laminate structure facilitated by bioinspired brick‐and‐mortar design using Bacterial Cellulose, Graphene Oxide (GO), and MXene demonstrates the potential of fabricating a thin film as moisture‐sensitive strip‐like actuator. The path of humidity absorption is guided by the maze‐like channel thanks to the parallel 2D‐platelet GO and MXene. Such time‐dependent slow diffusion causes the asymmetry of absorption as well as strain mismatch for morphing, whilst the thin structure enables the actuator to react sensitively to even trivial moisture, such as that from an approaching finger. Those materials thus require hydrophilic microstructures with channels to facilitate capillary action for water absorption by chemical potential gradients. Based on absorption, pH‐sensitive matrices can furtherly exchange protons in response to changes in environmental pH to induce more morphing (Figure 9C). Those actuators made by hydrogel networks contain ionizable groups (e.g., carboxylic acids, amines) that protonise or deprotonise to tune the density of electrons. Either way is designed for enhancing repelling force amongst chains and hydrophilicity, resulting in higher osmotic pressure for water molecules to enter for swelling.^[^ ^104^, ^110^ ^]^ For example, pine‐cone‐like bilayer hydrogel actuators composed by Poly(N‐isopropylacrylamide)‐ Acrylic Acid‐ Poly(diallyldimethylammonium chloride (PNIPAM‐AAc‐PDADMAC) can deprotonate by acrylic acid when pH is 6.5 to attract the PDADMAC. The resulting shrinkage can be assisted by a passive polydimethylsiloxane layer to generate the curvature toward one side. When pH decreases to 3, the protonation of acrylic acid can recover, and the hydrophilic PDADMAC can absorb water for swelling, inducing new curvature toward the other side. This system responds to both humidity and pH, enabling it to morph toward either sides.

While osmotic‐driven actuators exhibit large morphing capability, they are limited by the gradual water diffusion governed by Fick's law, which requires a long actuation time compared to bistability. For example, Ni et al. developed a hydrogel network where solvent desorption occurs above 90°C, but the slow diffusion specifically designs the 10 min delay morphing mechanism.^[^ ^111^ ^]^ However, the capillary effect, which helps the diffusion, permits the thickness of the actuator to be higher than other stimuli. Low actuation stress arises from low stiffness of matrix, inducing oriented fillers as reinforcement can enhance both the elastic modulus as well as toughness, while also tuning the anisotropic swelling ratio. Pores with gradient distribution can also be employed for matrix to deform larger. Specifically, ionizable functional groups like ‐COOH or ‐NH_2_ should be grafted on hydrogel network chains for pH‐sensitive purposes, which react when pH is around their pKa. Candidates can thus be polyacrylic acid, sodium alginate or chitosan.^[^ ^104^, ^110^, ^112^, ^113^ ^]^ It is also noticeable that the non‐toxicity of hydrogel paves way for applications in the medical field, and those self‐adaptive hydrogel actuators can potentially react to the human in vivo environment spontaneously.

Field‐based actuators can morph upon a change in temperature, light or magnetic field. All those energy sources can be transformed into kinetic energy by inducing microstructures with geometrical designs, whereas light and magnetic fields can also be converted to thermal energy as an intermediate step. Thermo‐responsive actuators achieve deformation by exploiting the strain mismatch caused by anisotropy in the coefficient of thermal expansion (CTE) upon global temperature change (Figure 9D). Design strategies typically involve inducing alignment of fiber with asymmetrical lay‐up design for laminate, or compositional, porosity gradients to generate anisotropic internal stresses upon heating or cooling. Polymeric materials generally show a large CTE, thus enabling a wide range of matrix choices.

Apart from composites or porous actuators, the matrix material itself can also show alignment on the molecular scale. For instance, extrusion‐based 3D printing can deposit LCE mesogen with alignment by shear and elongation stress. Combined with UV curing method, a gradient distribution of mesogen can also be created in the through‐thickness direction, which programs the function of morphing upon heating. LCE can then show reversible curling and high weightlifting as well as morphing toward complex 3D shapes. Indeed, LCE provides high actuation stress, which also offers the possibility of using 3D printing for inducing alignment and depositing layers for lamination inspired by nature rather than conventional casting with complex alignment control.^[^ ^114^, ^115^ ^]^

Meanwhile, hydrogels can also exhibit morphing upon phase separation during heating.^[^ ^106^, ^116^ ^]^ For example, Poly(N‐isopropylacrylamide) (PNIPAM) can tune the hydrophilicity of polymer chains upon changing temperature. The chain network swells when it is hydrophilic at temperatures lower than the lower critical solution temperature (LCST), which is usually around 32 °C, yet desorb water when it is hydrophobic at temperatures higher than the LCST. In this case, Tunicate Cellulose Nanocrystals as fillers also show alignment and gradient distribution, hence aiding the matrix to show strain mismatch for morphing. Shape‐memory polymers like Polylactic Acid or Polyurethane (PU) are also sensitive to temperature and can be accurately controlled. But their irreversible morphing nature limits them to self‐deploying applications, including deployment of folded solar panels in space as well as medical devices like stents during in‐plant surgeries. These thermal‐responsive actuators are expected to show uniform heating upon global temperature change within a short actuation time, which requires a low thickness design. Meanwhile, polymers show softening and degradation at high temperatures, while some phase‐separating hydrogel like Poly(acrylic acid)‐Calcium Acetate shows stiffening during heating. Therefore, the change of stiffness should also be considered with the tuning of temperature for a more accurate prediction of actuation stress in real cases.

Other stimuli like light or magnetic field can also be used to trigger morphing. Lights like UV can irradiate the azobenzene‐based liquid crystal polymers (LCPs) to induce trans‐to‐cis isomerization and realignment to drive morphing,^[^ ^117^ ^]^ but it can also induce rise of temperature (Figure 9E). MXenes can convert energy from NIR to heat, which increases the temperature of hydrogels like PNIPAM above LCST for shrinkage and morphing. Other light‐sensitive particles like CNTs^116^ and gold nanorods, can also generate heat through photothermal coupling, inducing anisotropic thermal expansion or shrinkage. Apart from choosing a matrix with high CTE, other fillers showing high thermal conductivity like graphene oxide (GO)^[^ ^106^ ^]^ can also be induced to enhance the efficiency of energy converting and morphing. Chiral dopants can also induce helical structures in LCEs for twisting motions.^[^ ^118^ ^]^ These actuators offer multi‐modal functionality (visible, NIR, UV light, and heat), rapid response like photothermal actuation, and non‐contact control. However, limitations include energy conversion efficiency from light to mechanical work (photomechanical coupling induces dissipating heat), potential thermal degradation, and limited light penetration depth restricting the effective actuation volume in thicker films. Those limitations require light‐responsive actuator to be transparent and thin.

Although magnetic‐responsive actuators can macroscopically generate magnetic force and torques by embedded fillers when exposed to a magnetic field for complex deformation,^[^ ^107^ ^]^ it is also noticeable that a matrix with magnetic particles can also generate heat under an alternative magnetic field (AMF) by Neel relaxation (Figure 9F).^[^ ^119^ ^]^ For example, Fe_3_O_4_ particles can react to AMF to heat up the surrounding PNIPAM for inducing thermal shrinkage above LCST, and the passive layer can restrict the shrinkage to generate curvature. In this design, morphing only occurs on the hinges of the actuator, indicating the potential in origami‐like actuator design with sufficient actuation stress, but the process requires a long actuation time. This mechanism also paves way for actuating in a dry environment under room temperature.

**Figure 10:**
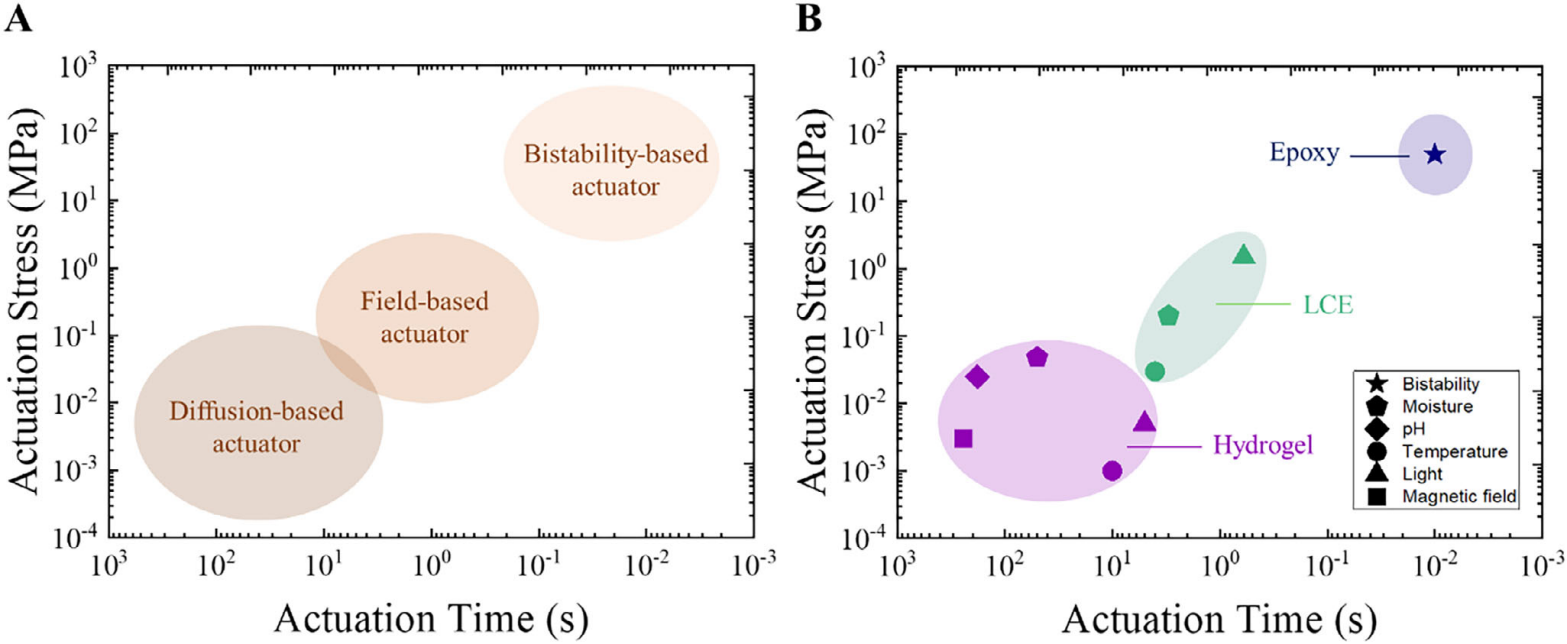
Performance comparison of bioinspired mechanisms of sensing‐actuation coupling with categories of triggering stimuli. **A)** Theoretical comparison of actuation stress versus actuation time based on the mechanism of actuation. (Note that the boundaries are merely illustrative and not derived from quantitative data. They should not be interpreted as strict limits on the applicable ranges of each technology) **B)** Empirical comparison of actuation stress versus actuation time provided by references. All the raw data are available in Table S3 (Supporting Information), but only representative data points are shown.

A theoretical classification of actuators based on their stimulus suggests the following performance: bistable systems are the fastest, followed by field‐based and then diffusion‐based mechanisms (Figure **10**A). However, a comprehensive study of the literature reveals convoluted results and only indicates the superiority of high stress and short time of bistable samples. Observing a larger dataset indeed reveals that the type of matrix largely determines the performance (Figure 10B).

The choice of polymer matrix, as such, is crucial to the ultimate performance of actuators. For instance, thermosets like epoxy provide high stiffness enabling high actuation stress in bistable configurations, but their brittleness and insensitivity to humidity limit their application. Hydrogels are highly versatile due to their sensitivity to a wide range of stimuli, yet applications are limited by slow diffusion and low stiffness. While filler reinforcement enhances stiffness, it often compromises the swelling ratio. LCEs offer a combination of high stiffness and exceptional sensitivity by molecular‐level programming, which allows for complex, pre‐programmed motions like twisting and bending. Figure 10B concludes that the hydrogel‐based actuators generally show low stress with slow morphing, followed by LCE, whilst epoxy‐based actuator shows high stress and short actuation time. Other matrices, though not commonly shown in literature, also exhibit potential for use as bioinspired actuators. Shape Memory Polymers (SMPs) excel in irreversible deformations and high‐force deployment actions. Although their processing requires a full thermo‐mechanical cycle, their shape‐memory effect is stable and accurate. Elastomers like PDMS and PU offer a balance between elasticity and durability, making them ideal for cyclic applications or as passive layers in laminates. Their processability and relatively low cost make them ideal for 3D printing, but low intrinsic stiffness limits their actuation stress compared to epoxy. Other emerging materials, like vitrimers with dynamic covalent bonds, also show potential shape‐morphing effect.^[^ ^120^ ^]^ Other factors including microstructures and geometry, also collectively determine the performance of actuators. The design process should therefore be application‐driven, selecting the optimal material system for niche markets such as soft robotics and biomedical devices.

Despite promising demonstrations, several fundamental challenges impede the practical application of these actuators. The lack of standardized testing protocols makes objective comparison amongst studies unreliable, which obscures the novel designs in industry. Crucial metrics like energy conversion efficiency are also rarely tested and reported. Meanwhile, long‐term durability remains a major issue. The inherent viscoelasticity causes energy dissipation and challenges long‐term durability due to time‐dependent creep and fatigue. Many actuators fail to withstand a few thousand cycles, which is insufficient for most real applications. Enhancing durability requires a wide range of approaches, including better surface treatment, eliminating stress concentrations through intelligent design, and improving interfacial bonding.

To overcome these barriers, designers should adopt standardized evaluation methods and harness computational tools like finite element method and machine learning for prediction, cooperating with advanced manufacturing techniques. This integrated approach is essential to accelerate the design of robust and reliable actuators for their widespread technological impact.

### 4.2 Toward Computation

**Figure 11:**
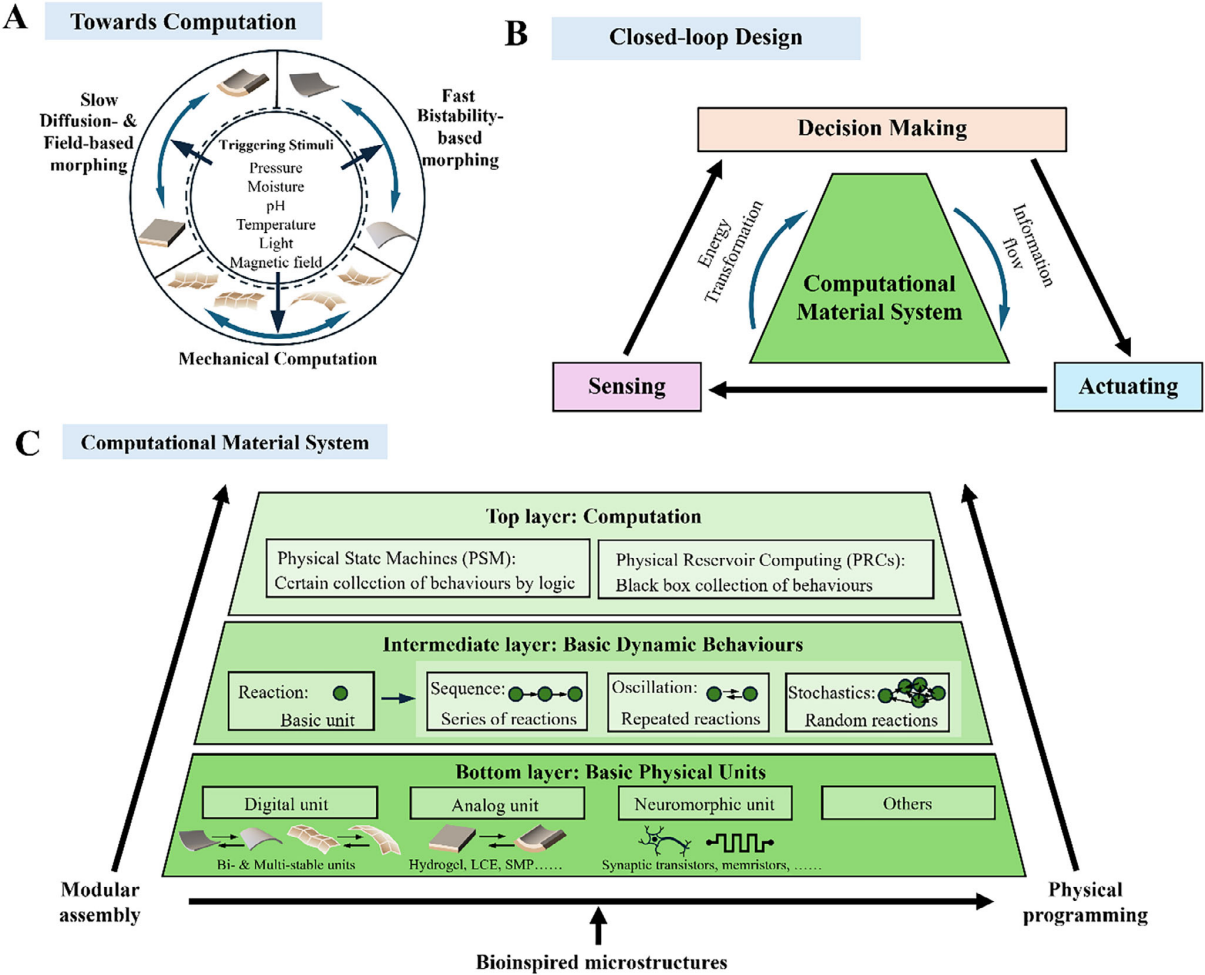
Schematics of concepts and designs of computational robotic materials. **A)** Simple sensing‐actuating coupled system (bistability‐, diffusion‐ and field‐based morphing mechanisms) showing potential for advancing toward computational actuators with embodied intelligence. **B)** Closed‐loop design of computational actuators with sensing‐decision making‐actuating loop aided by computational material system. The core of the loop is to design systems with the capability of energy transformation and information flow. **C)** Schematics of the computational material system are shown in **(B)**. Hierarchical design philosophy of computational systems comprises basic physical units, basic dynamic behaviors, and computation. Bioinspired microstructures help the evolution of building the actuator from modular assembly to physical programming.

Stimuli‐responsive composites shown in Section 4.1 can alter their shape in response to environmental cues, yet their functionality is typically confined to simple, pre‐programmed transformations, restricting their application in scenarios requiring adaptability and autonomy. To address this research gap, a new paradigm is emerging focused on embedding computational capabilities directly within the physical structure of materials (Figure **11**A).

The closed‐loop design comprises sensing, decision making, and actuation and is founded on two complementary first principles (Figure 11B), namely energy transformation and information flow. The former is defined by the intrinsic properties of materials and describes its energy transformation approach with potential states and behaviors, exhibited by actuation toward those stimuli demonstrated in Section 4.1. The second principle defines the input/output (I/O) architecture used to access and control the material's state. Its importance lies in enabling functional versatility, as altering the I/O architecture can reconfigure the same physical system for diverse computational tasks.^[^ ^121^ ^]^ Those two principles define how materials process energy and information upon sensing for actuation via decision‐making and feedback, which intrinsically form the closed‐loop system.

The construction of integrated computational materials can then be understood through a hierarchical framework based on the flow of energy and information (Figure 11C). The bottom layer of this framework consists of basic physical units as the building blocks of material intelligence. These units include digital units, such as bistable or multistable elements that serve as physical bits for storing information and constructing logic gates, as exemplified by mechanical bits inspired by an earwig wing or complex mechanical interfaces.^[^ ^109^, ^122^, ^123^, ^124^ ^]^ Also included are analog units, such as LCEs, hydrogels, and SMPs demonstrated in Section 4.1, which respond gradually to external stimuli to perform analog computation based on physical laws. Furthermore, neuromorphic units such as the synaptic transistors and memristors,^[^ ^125^, ^126^ ^]^ merge sensing, memory, and signal processing capabilities at the single‐device level. Other high‐performance actuation units such as the ethanol‐silicone composite with its exceptionally large 900% strain capability, provide a powerful physical basis for the system's output.^[^ ^127^ ^]^

These foundational units give rise to the intermediate layer of the framework: basic dynamic behaviors, which constitute the elementary computational operations by basic units from the first layer. Milana et al. identified three main elements^[^ ^128^ ^]^: the reaction, as a single jump between energy valleys that forms the basis of logic operations; the sequence, a pre‐programmed series of reactions, as demonstrated by 4D‐printed LCE strips,^[^ ^129^ ^]^ which execute complex bending patterns based on encoded printing speeds, the oscillation, a self‐sustained rhythmic behavior that can act as an internal physical clock (Figure 11C).^[^ ^128^ ^]^ The category can also include stochastic dynamics, which harnesses random processes like thermal motion as a computational resource. For example, the geometrically confined Brownian motion of a single skyrmion could be used to solve non‐linearly separable problems.^[^ ^130^ ^]^

By composing these dynamic primitives, the computational and control core can then be built as the top layer shown (Figure 11C). This layer enables complex information processing through approaches like deterministic computation via Physical State Machines (PSMs), where networked bistable elements form logic circuits. A more versatile paradigm is Physical Reservoir Computing (PRCs), which utilizes complex, high‐dimensional dynamics of a physical system itself as a computational resource. The core principle of PRC is to treat the material as a fixed, non‐linear reservoir as a black box that transforms input signals, requiring only a simple linear readout layer to be trained. The example of a skyrmion can also be considered as a reservoir to solve non‐linearly separable problems.^[^ ^130^ ^]^

Ultimately, these hierarchical layers enable the completion of the closed loop, representing the intelligent interaction of the material with its environment. This cycle begins with neuromorphic sensing as its input, which encodes physical quantities into bioinspired spike‐based signals.^[^ ^131^, ^132^ ^]^ The cycle culminates in physical actuation and bio‐interfaces as outputs, where computational results drive physical motion^[^ ^122^ ^]^ or communicate with biological systems.^[^ ^133^ ^]^ A notable prototype of this complete cycle is the artificial somatic reflex, which integrates a flexible sensor, a synaptic transistor, and an artificial muscle to demonstrate a full, intrinsic closed‐loop reflex where a sensory input above a threshold directly triggers physical actuation.^[^ ^125^ ^]^

While this framework outlines a compelling blueprint, translating the vision into material reality presents significant challenges centered on fabrication and energy efficiency. Conventionally, building such complex material systems requires modular assembly, which integrates discrete functional components.^[^ ^125^, ^133^ ^]^ This approach benefits from mature fabrication techniques but the interfaces between modules can limit overall flexibility and robustness. This limitation necessitates a move toward physical programming as a more integrated, monolithic pathway by encoding function directly into the matter during its fabrication.^[^ ^129^ ^]^ In this approach, bioinspired microstructures play a critical role as the primary tool for embedding intelligence. Nature provides a diverse range of design principles for creating materials with integrated functions through evolution, like brick‐and‐mortar with interlocking interface design^[^ ^134^ ^]^ as well as autonomous arms of an octopus. By mimicking these designs, materials can be developed that go beyond simple stimulus‐response. Furthermore, multi‐modal responsiveness can be achieved by integrating microstructures sensitive to different stimuli, such as combining humidity‐responsive hydrogels with temperature‐ or light‐responsive composites containing fillers like MXenes or carbon nanotubes. These examples demonstrate that bioinspired microstructures provide a powerful pathway to realize lightweighting and miniaturization, replacing bulky complexity with material‐level intelligence.

However, energy efficiency remains a critical barrier. Traditional actuators, though efficient, require bulky external controls that prevent miniaturization based on electricity. In contrast, new actuators based on bioinspired microstructures achieve functional integration but suffer from low energy conversion efficiency, with some high‐strain systems reporting values as low as 0.2%–0.3%. This contrasts significantly with the highly efficient systems found in nature, underscoring the potential for further understanding and utilisation of principles from nature. Future progress can thus be made toward efficient energy transduction and integrating them with advanced manufacturing to realize the full potential of autonomous computational materials.

## 5 Conclusions 

Through the examples of microstructured sensors, actuators, and robotic materials inspired by nature, translating these biological concepts into robotic systems involves careful choice of materials, microstructuring, proper design of structural hierarchy from micro‐ to macro‐scale, and selecting suitable fabrication approaches. Apart from all these, mechanics for modelling these bioinspired structures is also an important consideration. The integration of efficient sensing along with precise mechanical motion remains a core scientific challenge and thus, this review collectively focuses on the performance of both sensors and actuators as well as coupling through microstructures within them. Bioinspired microstructural control and hierarchical material design, be it in sensing or actuating systems offer better functionality.

While many microstructured and bioinspired sensors are initially characterized at the material level, their integration into more complex robotic systems has been successful. Sensors can detect not just pressure magnitude, but also the direction of applied force. This is a critical capability for precise manipulation in robotics.^[^ ^29^ ^]^ Similarly, bending and torsional strains can be detected through the use of microstructures.^[^ ^135^ ^]^ Furthermore, bioinspired microstructured sensors can be used for sliding detection and object recognition.^[^ ^78^ ^]^

Bioinspired nacre‐like microstructured actuator can mimic a dragonfly‐shaped robotic under periodic humidity on and off.^[^ ^65^ ^]^ Also, the actuator can show the movements of a biomimetic human finger and manipulating artificial muscle for lifting in response to humidity. Fibrous actuator can function as a smart fan driven by water or moisture.^[^ ^77^ ^]^ Tubular soft composite actuator can operate as a passive solar tracker/ reflector.^[^ ^78^ ^]^ Several other grippers, micro rolling devices, and load carrying actuators have been developed using these bioinspired microstructured actuators.^[^ ^76^, ^80^ ^]^

As the field advances, incorporating bioinspired microstructured sensors and actuators into closed‐loop robotic systems could enhance perception and motion control, in next‐generation soft and bioinspired robots.

In robotic material, variations in microstructures and multilayer or gradient structures generate non‐uniform stress distributions, enabling complex deformations upon global stimuli. If finer and controllable microstructural design are achieved through advanced manufacturing especially with 3D and 4D printing, we can expect surpassed sensitivity, energy conversion efficiency, and actuation stress. While bioinspired microstructuring in sensors and actuators is not just limited to translate the idea of microstructuring found in biological materials to innovate. This should also include the potential of mimicking these biological structures with robust fabrication strategies. Finally, leveraging those microstructures enables the use of more diverse sources of feedstocks which bear lower environmental, ethical and social cost, for the design and fabrication of the next generation of sustainable robotic systems.

## 6 Outlook

Advancements in fabrication methods remain central to the transition of bioinspired sensors and actuators from laboratory prototypes to fully integrated, functional systems. While techniques such as laser patterning and photolithography offer precision, their limitations in scalability and durability restrict their broader applicability, particularly in the development of robust, long‐lifetime systems. Additive manufacturing approaches especially 3D and emerging 4D printing have enabled the construction of complex bioinspired architectures.^[^ ^136^, ^137^ ^]^

However, most demonstrations remain confined to structural components rather than complete, monolithic actuator assemblies. For these devices to be viable in real‐world robotic applications, fabrication strategies must evolve to accommodate high‐throughput, multi‐material processing with precise spatial control.^[^ ^138^ ^]^ One of the pathways should be addressing processing challenges associated with responsive materials like liquid crystal elastomers and other soft composites.

Emerging approaches such as artificial intelligence‐guided structural optimization also hold promise in rationalizing bioinspired designs for both function and manufacturability.^[^ ^139^ ^]^ As a final point, reimagining materials not just as passive carriers, but as active participants in logic and movement, is a path to move forward. By amplifying micro‐scale phenomena, encoding decisions in structural dynamics, and embedding intelligence in bistable and responsive forms, materials that sense multiple stimuli and act appropriately according to these stimuli could be designed. The convergence of scalable fabrication, material‐process compatibility, and structure‐function co‐design will be essential in realizing sensor‐actuator systems capable of closed‐loop control, adaptive response, and long‐term operation.

## Conflict of Interest

The authors declare no conflict of interest.

## Author Contributions

S. contributed to conceptualization, formal analysis, data curation, and visualization, and wrote the original Draft. R.P.B. contributed to formal analysis, data curation, and visualization, and wrote the original draft. H.Z. contributed to formal analysis, data curation, visualization, and wrote the original draft. Y.W. acquired funding, wrote, reviewed & edited the final manuscript. H.L.F. contributed to conceptualization, and supervision, wrote, reviewed & edited the final manuscript.

## Supporting information



Supporting Information
