# Supplementary material for: Robotic Materials With Bioinspired Microstructures for High Sensitivity and Fast Actuation
Source: Adv Sci (Weinh). 2025 Sep 25;13(15):e09739. doi: 10.1002/advs.202509739 (PMC13042804; doi:10.1002/advs.202509739)
Supplement: Supplementary file 1 — Supporting Information [file ADVS-13-e09739-s001.docx]

Supporting Information

**Microstructured Bioinspired Robotic Materials for High Sensitivity and Fast Actuation**

*Sakshi^a^, Rohit Pratyush Behera^a^, Hongyu Zhou^a^, Yifan Wang^a^ and Hortense Le Ferrand^a,b^**

^a^School of Mechanical and Aerospace Engineering, Nanyang Technological University Singapore, Singapore

^b^School of Material Science and Engineering, Nanyang Technological University Singapore, Singapore

*E-mail: hortense@ntu.edu.sg

**Table 1.** Microstructures with their corresponding fabrication strategy, sensor performance metrics (sensitivity along with their pressure range, time response and limit of detection).

| Type of Microstructure | Fabrication Strategy | Mechanism | Pressure Range (Low) kPa | Sensitivity kPa-1 | Pressure Range (High) | Sensitivity kPa-1 | Limit of Detection (Pa) | Response Time (ms) | Durability | Ref |  |
| --- | --- | --- | --- | --- | --- | --- | --- | --- | --- | --- | --- |
| Spinosum microstructure | Photolithography | Piezoresistive | 0–50 | 67.1 | 50-250 | 17.1 | 7 | 40 | 10000 | ^[1]^ |  |
| Spinosum microstructure | Laser direct writing | Piezoresistive | 0-13.5 | 68.3 | - | - | 68.6 | 90 | 10000 | ^[2]^ |  |
| Spinosum microstructure | Using Urchin-shaped microcapsules | Piezoresistive | 0–7 | 24.63 | - | - | 8 | 14 | 5000 | ^[3]^ |  |
| Cilia | Two-photon polymerization | Capacitive | 0-6 | 0.0513 | 6-120 | 0.0079 | 30 | 60 | 5000 | ^[4]^ |  |
| Cilia | Magnetic field | Piezoresistive | 0-0.1 | 4 | 0.1-1 | 0.25 | 0.9 | - | 10000 | ^[5]^ |  |
| Cilia | Magnetic field | Capacitive | 0–10 | 0.28 | 50–200 | 0.02 | 2 | 100 | 500 | ^[6]^ |  |
| HS-I | Laser direct Writing | Piezoresistive | 0-0.6 | 0.9 | 0.6-10 | 11.06 | - | - | 1000 | ^[7]^ |  |
| HS-I | Laser direct writing | Capacitive | 0-22 | 4.48 | 27–65 | 0.86 | 3 | 7 | 1000 | ^[8]^ |  |
| Lotus leaves/HS-I | Templating from lotus | Capacitive | 0-25 | 1.2 | - | - | 5 | - | 1000 | ^[9]^ |  |
| Lotus leaves/ HS-I | Templating from lotus | Capacitive | 0-2 | 1.2 | 2-12.0 | 0.077 | 0.8 | 36 | 100000 | ^[10]^ |  |
| Lotus leaves/HS-I | Templating from lotus | Piezoresistive | 0.3-0.4 | 0.58 | - | - | - | - | - | ^[11]^ |  |
| Lotus leaves/HS-I | Templating from lotus | Capacitive | 0-50N | 0.815 | - | - | 17.5 | 38 | - | ^[12]^ |  |
| Rose Petal/HS-I | Templating from rose |  | 1-1.5 | 0.145 | 1-10.0 | 0.06 | 30 | 13.4 | 7000 | ^[13]^ |  |
| Rose Petal/HS-I | Templating from rose | Capacitive | 0.5–10 | 0.055 | - | - | - | 200 | - | ^[14]^ |  |
| Rose Petal/HS-I | Templating from rose | Piezoresistive | 0-2 | 1.35 | 2.0-5.0 | 0.1 | 2 | 36 | 5000 | ^[15]^ |  |
| Rose Petal/HS-I | Templating from rose | Piezoresistive | 0-0.5 | 70 | 0.5-2 | 19 | 0.88 | 30 | 1000 | ^[16]^ |  |
| Rose Petal/HS-I | Templating from rose | Piezoresistive | 0.0584-0.96 | 53 | 0.96-5 | 1.03 | 58.4 | 38 | 50 000 | ^[17]^ |  |
| Gingko Leaf/HS-I | Templating from Gingko leaves | Piezoresistive | 0-15.0 | 5.9 | - | - | - | 42 | 2000 | ^[18]^ |  |
| Gingko Leaf/HS-I | Templating from Gingko leaves | Piezoresistive | 0-10 | 164.93 | 10.0-18.0 | 403.46 | 0.88 | 99.3 | 12000 | ^[19]^ |  |
| Gingko Leaf/HS-I | Templating from Gingko Leaf | Capacitive | 0-1 | 1.194 | 50-300 | 0.0086 | 6.53 | 80 | - | ^[20]^ |  |
| Mimosa Leaf/HS-I | Templating from Mimosa leaves | Piezoresistive | 0-0.07 | 50.17 | 0.2-1.5 | 1.38 | - | 20 | 10000 | ^[21]^ |  |
| Epipremnum Aureum/HS-I | Templating from EA leaves | Piezoresistive | 0-0.3 | 19.8 | 0.3-6 | 0.27 | 0.6 | - | 35 000 | ^[22]^ |  |
| Epipremnum Aureum/HS-I | Templating from EA leaves | Piezoresistive | 0-0.14 | 83.9 | 0.14-10 | 0.4 | 0.5 | 90 | 28000 | ^[23]^ |  |
| Epipremnum Aureum/HS-I | Templating from EA leaves | Piezoresistive | 0-0.2 | 110 | 0.2-15 | 3 | 0.2 | 30 | 10000 | ^[24]^ |  |
| Calathea Zebrine/HS-I | Templating from Calathea Zebrine | Capacitive | 0-0.5 | 54.31 | 0.5-10 | 30.11 | - | 29 | 5400 | ^[25]^ |  |
| HS-II | Lithography/Micro-engraving | Capacitive | 0-1700 | 0.065 | - | - | - | 100 | 7000 | ^[26]^ |  |
| Micropyramid/HS-II | Photolithography and anisotropically wet etching | Capacitive | - | 3.73 | - | - | 0.1 | 21 | 5000 | ^[27]^ |  |
| Microdome/HS-II | Lithography after Water Droplet Spray | Piezoresistive | 0-0.2 | 124 | 0.2-5.0 | 0.39 | 2 | - | 1200 | ^[28]^ |  |
| Microdome/HS-II | Thermal Treatment and internal popping | Piezoresistive | 0-0.05 | 50.45 | 0.05-0.4 | 4.35 | 0.209 | 39 | 4000 | ^[29]^ |  |
| Micro-protuberances/HS-II | Sand Paper Templating | Capacitive | 0.1-11 | 0.18 | - | - | 100 | 56.3 | 3000 | ^[30]^ |  |
| Micropyramid/CS | Photolithography | Capacitive | 0-2 | 0.55 | 2-7 | 0.15 | 3 | - | 10000 | ^[31]^ |  |
| Micropyramid/CS | Photolithography/ Etching | Capacitive | 0-0.05 | 70.6 | 0.05-0.35 | 3.3 | 1 | - | 2000 | ^[32]^ |  |
| Micropillar (tilted) | Mask photoresist | Capacitance | 0-1.5 | 0.42 | 1.5-5 | 0.04 | 100 | 1 | 1000 | ^[33]^ |  |
| Micropillar+fiber | Lithography/Electrospinning | Capacitive | 0-7 | 0.6 | 7.0-15.0 | 0.51 | 0.065 | 25 | 10000 | ^[34]^ |  |
| Micropillar/CS | Photolithography | Capacitive | 0-1 | 0.43 | - | - | 3.4 | 33 | 1000 | ^[35]^ |  |
| Micropyramid/CS | Lithography | Piezoresistive | 0-0.1 | 5.53 | 0.1-1.4 | 0.1 | 2 | 0.2 | 5000 | ^[36]^ |  |
| Micropyramid/CS | Lithography | Piezoresistive | 0.37-5.9 | 4.88 | - | - | 37 | 0.2 | 800 | ^[37]^ |  |
| Micropyramid/CS | Photolithography/Wet Etching | Piezoresistive | 0-0.1 | 9.95 | - | - | - | 200 | - | ^[38]^ |  |
| Micropyramid/CS | Lithography | Piezoresistive | 0-0.1 | 1907.2 | 0.1-1 | 461.5 | 0.075 | 0.05 | 15000 | ^[39]^ |  |
| Micropyramid/CS | Photolithography/Etching | Piezoresistive | 0-0.6 | 23 | 0.6-3.0 | 0.7 | - | 10 | 10000 | ^[40]^ |  |
| Microcone/CS | Photolithography/ Laser Engraved Mold | Piezoresistive | 0-0.16 | 2.5 | 0.16-0.5 | 0.2 | 15 | 20 | - | ^[41]^ |  |
| Microdome/CS | Lithography/ Fabricated PS Spherical Array | Piezoresistive | 0-0.1 | 15 | 0.1-0.4 | 2 | 4 | 100 | 1000 | ^[42]^ |  |
| Microdome/CS | Laser Microstructuring | Piezoresistive | 0-2 | 1.82 | - | - | 1 | 36 | 6000 | ^[43]^ |  |
| Microdome/CS | Lithography/ Fabricated PS Spherical Array | Capacitive | 0-0.13 | 30.2 | 0.13-10 | 0.47 | 0.7 | 25 | 100000 | ^[44]^ |  |

^a)^The durability of a sensor (N) refers to the number of loading/unloading cycles (N) before which there are negligible changes in capacitance and resistance.

**Table 2.** Microstructures with their corresponding material, actuator performance metrics (response time, generated stress, durability) along with the stimulating mechanism and maximum possible size of the actuators.

| Micorstructure | Materials | Response time (s) | Generated stress  (MPa) | Durability (cycles) | Work capacity (J/Kg) | Stimulation | Size | Ref |
| --- | --- | --- | --- | --- | --- | --- | --- | --- |
| Nacre-like | GO + Chitosan | 16 | 66.5 | 1000 | 6.17 | Moisture | 9.87 | ^[45]^ |
| Nacre-like | carbon nanomeshes, PVDF, EMIM-BF4 | 1.5 | 23 | 10000 | - | Electrical | 24.75 | ^[46]^ |
| Nacre-like | CMC, MXene nanosheets, Al3+ | 2.3 | 273.6 | 1500 | 98.06 | Moisture | 3.6 | ^[47]^ |
| Nacre-like | Tensylon HSBD30A, UHMWPE | - | 119 | - | - | Mechanical cutting | - | ^[48]^ |
| Nacre-like with gradient | Mxene, epoxidised natural rubber | 2.4 | 25.03 | - | 46296 | Thermal | - | ^[49]^ |
| Turing pattern | urethane acrylates, isobornyl acrylate, 2-hydroxyethyl acrylate | - | 0.323 | - | 59.33 | Mechanical pressure | 335714 | ^[50]^ |
| Venus fly trap inspired | Alumina + Ni flakes + epoxy + hydrogel | 0.05 | 140 | - | - | Mechanical pressure | 10000 | ^[51]^ |
| Geiko-inspired lamellar | PDMS, crosslinker, CIP | 0.1 | - | - | - | Magnetic | 1267.2 | ^[52]^ |
| Layered | OSA/PAAm/PPy/LiCl | - | 0.86 | - | 2430.258 | photo-thermal | 45 | ^[53]^ |
| Layered | Bi0.5Na0.5TiO3-based ceramics | 100 | 0.18 | 1000 | 75.11 | Electric | 119 | ^[54]^ |
| Janus | Ti3C2Tx + CNF + PE | 6.8 | 32.5 | - | - | Light, electricity & humidity | 1.05 | ^[55]^ |
| Fabry−Pérot Microcavity Architecture | Ti3AlC2 Mxene + PC | 10 | - | 1000 | - | electric | 0.036 | ^[56]^ |
| Muscle-like fibrous | silicone rubber | 0.5 | 2.36 | - | 14.35 | Fluid pressure | 178500 | ^[57]^ |
| helicoidal | CMC fibers | 0.3 | 27 | 150 | 1962 | humidity | 2.57 | ^[58]^ |
| helicoidal | LCE, LM, Mecoflex | 14 | 0.0025 | 100 | 0.00623 | Thermal | - | ^[59]^ |
| Helicoidal | BC + LCE | 2.5 | 43.9 | 5000 | - | Thermal | - | ^[60]^ |
| Gradient | PNIPAM + PAAm | 9 | 0.32 | 272 | - | Thermal | 2100 | ^[61]^ |
| Tubular | PU | 100 | 0.24 | 100 | 240 | Moisture | 1110 | ^[62]^ |
| Tubular | PU | 120 | 0.6 | 120 | 600 | Hydrothermal | 1110 | ^[62]^ |
| Janus | RGO + CLCE | 18 | 1.4 | 100 | - | Thermal | 256 | ^[63]^ |
| Tubular | LCE | 30 | 0.35 | 100 | 150 | - | 1170 | ^[64]^ |
| Mushroom | PDMS + MWCNTs | 5 | 0.00586 | - | 0.0107 | Light | 1.625 | ^[65]^ |
| Heliciodal and tubular | UHMWPE + PP | - | 65 | 1000 | 0.00318 | Mechanical tensile stress | 3.801 | ^[66]^ |
| Fiber-based muscles | PE + COCe | 4 | 10 | 100000 | 0.1855 | thermal | 0.0005 | ^[67]^ |
| Dual gradient | rGO + PDMAEMA | 1 | - | - | 0.4 | UV light | - | ^[68]^ |
| Fiber alignment gradient | PVA | 0.15 | 0.0012 | 1000 | 10.9 | Moisture | 120 | ^[69]^ |
| Gradient hydrogel | PEG600DA, HEMA, AUD, Irgacure 819, Orasol dye, SPMA | 35 | - | - | - | water | 140.4 | ^[70]^ |
| Octopus tentacle inspired gradient | NIPAM + Ag flakes | 8 | 0.0252 | 50 | 78.48 | thermal | 700 | ^[71]^ |

**Table 3.** Actuator performance metrics (response time, stress, thickness) with categories of triggering stimuli.

| Stimuli | Actuation Time | Actuation Time (shown in graph)  Unit: s | Actuation Stress | Actuation Stress (shown in graph)  Unit: MPa | Thickness | Ref | | |
| --- | --- | --- | --- | --- | --- | --- | --- | --- |
| Bistability | 10 ms - 200 ms | 0.01 | 0.6 Mpa | 0.6 | 0.4 mm | ^[72]^ | | |
| Bistability | 50 ms | 0.05 | 140^a^ Mpa | 140 | 0.8 mm | ^[51]^ | | |
| Bistability | 10 ms - 250 ms | 0.01 | 35 – 70^a^ Mpa | 50 | 0.35 mm | ^[73]^ | | |
| Bistability | 200 - 300 ms | 0.2 | 1.2-2 Mpa | 1.5 | 0.95 mm | ^[74]^ | | |
|  |  |  |  |  |  |  | | |
| Moisture | 10 seconds | 10 | 25 kPa | 0.025 | 0.5 mm | ^[75]^ | | |
| Moisture | 44 - 60 seconds | 50 | 47 - 50 kPa | 0.048 | 0.1 mm | ^[76]^ | | |
| Moisture | 80 seconds | 80 | 27 Mpa | 27 | 27 micron | ^[77]^ | | |
| Moisture | 1 - 5 seconds | 3 | 0.1 - 0.3^a^ Mpa | 0.2 | 4 15 25 micron | ^[78]^ | | |
| Moisture | 0.6 - 1.2 seconds | 1 | 25 - 65 kPa | 0.04 | 19 micron | ^[79]^ | | |
| Moisture | 40-60 seconds | 50 | 0.1 - 0.3 Mpa | 0.2 | 25 micron | ^[80]^ | | |
| Moisture | 10-30 minutes | 1200 | 2-5 kPa | 0.0035 | 0.5 mm | ^[81]^ | | |
| Moisture | 4 seconds | 4 | 0.45 Mpa | 0.45 | 10 micron | ^[82]^ | | |
| Moisture | 6-24 hours | 36000 | 0.5-2^a^ kPa | 0.001 | 0.8 mm | ^[83]^ | | |
| Moisture | 14 - 60 seconds | 40 | 0.3 kPa | 0.0003 | 0.14 mm | ^[84]^ | | |
|  |  |  |  |  |  |  | | |
| pH | 10 minutes | 600 | 30-50^a^ kPa | 0.04 | 1.5 mm | ^[85]^ | | |
| pH | 8-200 minutes | 6000 | 3-10^a^ kPa | 0.007 | 1 mm | ^[86]^ | | |
| pH | ~10 seconds | 10 | 0.23^a^ MPa | 0.23 | 0.4 mm | ^[87]^ | | |
| pH | 10 minutes | 600 | 0.1 - 2.5^a^ kPa | 0.001 | 0.5 mm | ^[88]^ | | |
| pH | 1-5 minutes | 180 | 25 kPa | 0.025 | 0.5 mm | ^[75]^ | | |
| pH | 15 seconds | 15 | 3.5^a^ kPa | 0.0035 | 1 mm | ^[89]^ | | |
|  |  |  |  |  |  |  | | |
|  |  |  |  |  |  |  | | |
| Temperature | 2 seconds | 2 | 0.23^a^ MPa | 0.23 | 0.4 mm | ^[87]^ | | |
| Temperature | 1 minute | 60 | 0.1 - 2.5^a^ kPa | 0.002 | 0.5 mm | ^[88]^ | | |
| Temperature | 10 seconds | 10 | 6-8^a^ kPa | 5.00E-04 | 1 mm | ^[90]^ | | |
| Temperature | 3 seconds | 3 | 4-6^a^ kPa | 5.00E-04 | 1 mm | ^[91]^ | | |
| Temperature | 3-5 seconds | 4 | 25-35^a^ kPa | 0.03 | 30 micron | ^[92]^ | | |
| Temperature | 10 seconds | 10 | 0.5 – 1^a^ kPa | 0.001 | 1 mm | ^[93]^ | | |
| Temperature | 0.1 seconds | 0.1 | 310^a^ kPa | 0.31 | 50 micron | ^[94]^ | | |
| Temperature | 1-3 minutes | 120 | 1-3^a^ kPa | 0.002 | 0.8 mm | ^[83]^ | | |
|  |  |  |  |  |  |  | | |
| Light | 2-100 seconds | 50 | 0.1 - 2.5^a^ kPa | 0.002 | 0.5 mm | ^[88]^ | | |
| Light | 5 seconds | 5 | 4-6^a^ kPa | 0.005 | 1 mm | ^[91]^ | | |
| Light | 2-3 seconds | 2.5 | 8-10^a^ kPa | 0.009 | 75 micron | ^[95]^ | | |
| Light | 0.6 seconds | 0.6 | 1.5 Mpa | 1.5 | 1 mm | ^[96]^ | | |
| Light | 1-3 seconds | 2 | 1-3 Mpa | 2 | 30 micron | ^[97]^ | | |
| Light | 30 seconds | 30 | 5^a^ kPa | 0.005 | 0.6 mm | ^[98]^ | | |
| Light | 1-2 second | 1.5 | 1-1.5^a^ kPa | 0.0015 | 2 mm | ^[99]^ | | |
| Light | 1-2 minutes | 75 | 50-120^a^ kPa | 0.08 | 50 micron | ^[100]^ | | |
| Light | 12-32 seconds | 20 | 0.2 kPa | 2.00E-04 | 0.14 mm | ^[84]^ | | |
|  |  |  |  |  |  |  | | |
|  |  |  |  |  |  |  |  |  |

^a)^ value was not directly given, calculated using simple Hooke's law.

[1] S. Tu, Y. Xi, X. Cui, Z. Xu, Z. Liu, Y. Zhu, *Chemical Engineering Journal* **2024**, *496*, 154083.

[2] Q. Zhou, B. Ji, G. Chen, Y. Ding, J. Wu, J. She, S. Wang, B. Zhou, *ACS Appl Nano Mater* **2019**, *2*, 7178.

[3] K. Wang, Z. Lou, L. Wang, L. Zhao, S. Zhao, D. Wang, W. Han, K. Jiang, G. Shen, *ACS Nano* **2019**, *13*, 9139.

[4] H. Yu, H. Guo, J. Wang, T. Zhao, W. Zou, P. Zhou, Z. Xu, Y. Zhang, J. Zheng, Y. Zhong, X. Wang, L. Liu, *Advanced Science* **2024**, *11*, 2305883.

[5] Y.-F. Liu, Y.-F. Fu, Y.-Q. Li, P. Huang, C.-H. Xu, N. Hu, S.-Y. Fu, *J Mater Chem B* **2018**, *6*, 896.

[6] Q. Zhou, B. Ji, Y. Wei, B. Hu, Y. Gao, Q. Xu, J. Zhou, B. Zhou, *J Mater Chem A Mater* **2019**, *7*, 27334.

[7] C. Zhang, R. Chen, C. Xiao, H. Zhao, Y. Wang, D. Geng, S. Chen, T. Luo, W. Zhou, *Adv Mater Interfaces* **2022**, *9*, 2101596.

[8] Q. Du, L. Liu, R. Tang, J. Ai, Z. Wang, Q. Fu, C. Li, Y. Chen, X. Feng, *Adv Mater Technol* **2021**, *6*, 2100122.

[9] J. Shi, L. Wang, Z. Dai, L. Zhao, M. Du, H. Li, Y. Fang, *Small* **2018**, *14*, 1800819.

[10] Y. Wan, Z. Qiu, Y. Hong, Y. Wang, J. Zhang, Q. Liu, Z. Wu, C. F. Guo, *Adv Electron Mater* **2018**, *4*, 1700586.

[11] D. Wang, X. Zhou, R. Song, C. Fang, Z. Wang, C. Wang, Y. Huang, *Chemical Engineering Journal* **2021**, *404*, 126940.

[12] T. Li, H. Luo, L. Qin, X. Wang, Z. Xiong, H. Ding, Y. Gu, Z. Liu, T. Zhang, *Small* **2016**, *12*, 5042.

[13] Z. Luo, J. Duan, H. Xu, Y. Wang, J. Liu, X. Yao, B. Zhang, *IEEE Sens J* **2021**, *21*, 20119.

[14] C. Mahata, H. Algadi, J. Lee, S. Kim, T. Lee, *Measurement* **2020**, *151*, 107095.

[15] Y. Wei, S. Chen, Y. Lin, Z. Yang, L. Liu, *J Mater Chem C Mater* **2015**, *3*, 9594.

[16] S. Yu, L. Li, J. Wang, E. Liu, J. Zhao, F. Xu, Y. Cao, C. Lu, *Adv Funct Mater* **2020**, *30*, 1907091.

[17] T. Yang, W. Deng, X. Chu, X. Wang, Y. Hu, X. Fan, J. Song, Y. Gao, B. Zhang, G. Tian, D. Xiong, S. Zhong, L. Tang, Y. Hu, W. Yang, *ACS Nano* **2021**, *15*, 11555.

[18] Y. Wang, W. Zhu, Y. Yu, P. Zhu, Q. Song, Y. Deng, *IEEE Sens J* **2020**, *20*, 7354.

[19] J. Yan, Y. Ma, G. Jia, S. Zhao, Y. Yue, F. Cheng, C. Zhang, M. Cao, Y. Xiong, P. Shen, Y. Gao, *Chemical Engineering Journal* **2022**, *431*, 133458.

[20] P. Zhang, J. Zhang, Y. Li, L. Huang, *J Phys D Appl Phys* **2021**, *54*, 465401.

[21] B. Su, S. Gong, Z. Ma, L. W. Yap, W. Cheng, *Small* **2015**, *11*, 1886.

[22] M. Jian, K. Xia, Q. Wang, Z. Yin, H. Wang, C. Wang, H. Xie, M. Zhang, Y. Zhang, *Adv Funct Mater* **2017**, *27*, 1606066.

[23] T. Zhao, T. Li, L. Chen, L. Yuan, X. Li, J. Zhang, *ACS Appl Mater Interfaces* **2019**, *11*, 29466.

[24] K. Xia, C. Wang, M. Jian, Q. Wang, Y. Zhang, *Nano Res* **2018**, *11*, 1124.

[25] Z. Qiu, Y. Wan, W. Zhou, J. Yang, J. Yang, J. Huang, J. Zhang, Q. Liu, S. Huang, N. Bai, Z. Wu, W. Hong, H. Wang, C. F. Guo, *Adv Funct Mater* **2018**, *28*, 1802343.

[26] B. Ji, Q. Zhou, M. Lei, S. Ding, Q. Song, Y. Gao, S. Li, Y. Xu, Y. Zhou, B. Zhou, *Small* **2021**, *17*, 2103312.

[27] W. Cheng, J. Wang, Z. Ma, K. Yan, Y. Wang, H. Wang, S. Li, Y. Li, L. Pan, Y. Shi, *IEEE Electron Device Letters* **2018**, *39*, 288.

[28] Z.-H. Tang, S.-S. Xue, Y.-Q. Li, Z.-C. Zhu, P. Huang, S.-Y. Fu, *ACS Appl Mater Interfaces* **2021**, *13*, 48009.

[29] Y. Jung, J. Choi, W. Lee, J. S. Ko, I. Park, H. Cho, *Adv Funct Mater* **2022**, *32*, 2201147.

[30] J. Huang, X. Tang, F. Wang, Z. Wang, Y. Niu, H. Wang, *Adv Eng Mater* **2022**, *24*, 2101767.

[31] S. C. B. Mannsfeld, B. C.-K. Tee, R. M. Stoltenberg, C. V. H.-H. Chen, S. Barman, B. V. O. Muir, A. N. Sokolov, C. Reese, Z. Bao, *Nat Mater* **2010**, *9*, 859.

[32] M. Li, J. Liang, X. Wang, M. Zhang, *Sensors* **2020**, *20*, 371.

[33] Y. Luo, J. Shao, S. Chen, X. Chen, H. Tian, X. Li, L. Wang, D. Wang, B. Lu, *ACS Appl Mater Interfaces* **2019**, *11*, 17796.

[34] M.-F. Lin, C. Cheng, C.-C. Yang, W.-T. Hsiao, C.-R. Yang, *Org Electron* **2021**, *98*, 106290.

[35] Z. Luo, J. Chen, Z. Zhu, L. Li, Y. Su, W. Tang, O. M. Omisore, L. Wang, H. Li, *ACS Appl Mater Interfaces* **2021**, *13*, 7635.

[36] B. Zhu, Z. Niu, H. Wang, W. R. Leow, H. Wang, Y. Li, L. Zheng, J. Wei, F. Huo, X. Chen, *Small* **2014**, *10*, 3625.

[37] C.-L. Choong, M.-B. Shim, B.-S. Lee, S. Jeon, D.-S. Ko, T.-H. Kang, J. Bae, S. H. Lee, K.-E. Byun, J. Im, Y. J. Jeong, C. E. Park, J.-J. Park, U.-I. Chung, *Advanced Materials* **2014**, *26*, 3451.

[38] X. Li, W. Huang, G. Yao, M. Gao, X. Wei, Z. Liu, H. Zhang, T. Gong, B. Yu, *Scr Mater* **2017**, *129*, 61.

[39] H. Li, K. Wu, Z. Xu, Z. Wang, Y. Meng, L. Li, *ACS Appl Mater Interfaces* **2018**, *10*, 20826.

[40] B. Zhu, Y. Ling, L. W. Yap, M. Yang, F. Lin, S. Gong, Y. Wang, T. An, Y. Zhao, W. Cheng, *ACS Appl Mater Interfaces* **2019**, *11*, 29014.

[41] A. dos Santos, N. Pinela, P. Alves, R. Santos, E. Fortunato, R. Martins, H. Águas, R. Igreja, *Adv Electron Mater* **2018**, *4*, 1800182.

[42] Y. Zhang, Y. Hu, P. Zhu, F. Han, Y. Zhu, R. Sun, C.-P. Wong, *ACS Appl Mater Interfaces* **2017**, *9*, 35968.

[43] Y. Gao, C. Lu, Y. Guohui, J. Sha, J. Tan, F. Xuan, *Nanotechnology* **2019**, *30*, 325502.

[44] Y. Xiong, Y. Shen, L. Tian, Y. Hu, P. Zhu, R. Sun, C.-P. Wong, *Nano Energy* **2020**, *70*, 104436.

[45] Y. Zhang, H. Jiang, F. Li, Y. Xia, Y. Lei, X. Jin, G. Zhang, H. Li, *J Mater Chem A Mater* **2017**, *5*, 14604.

[46] X. Han, M. Kong, M. Li, X. Li, W. Yang, C. Li, *J Mater Chem C Mater* **2020**, *8*, 1634.

[47] J. Wei, S. Jia, C. Ma, J. Guan, C. Yan, L. Zhao, Z. Shao, *Chemical Engineering Journal* **2023**, *451*.

[48] L. Jin, M. Yeager, Y.-J. Lee, D. J. O’Brien, S. Yang, *Sci Adv* **2022**, *8*, 3248.

[49] C. Yu, X. Li, X. Yang, X. Qiu, X. Zhang, Z. Chen, Y. Luo, *Small* **2024**, *20*.

[50] M. Tanaka, S. M. Montgomery, L. Yue, Y. Wei, Y. Song, T. Nomura, H. J. Qi, *Sci Adv* **2023**, *9*.

[51] H. Le Ferrand, K. S. Riley, A. F. Arrieta, *Bioinspir Biomim* **2022**, *17*.

[52] M. Li, Z. Yang, P. Wang, T. Wang, L. Shi, *Bioinspired Microplate Arrays for Magnetically Induced Anisotropic Solid Transport*.

[53] D. Bao, F. Guan, X. Ji, X. Zhang, Y. Xu, Q. Yang, Q. Yao, S. Zhang, J. Guo, *Chemical Engineering Journal* **2025**, *505*.

[54] D. Zhai, L. Zhao, Q. Zhang, C. Chen, S. Chen, D. Ju, X. Yuan, X. Zhou, D. Zhang, *Ceram Int* **2025**.

[55] Z. Song, T. Wu, L. Zhang, H. Song, *J Colloid Interface Sci* **2025**, *688*, 183.

[56] J. Wang, X. Guo, C. Li, H. Zhou, Y. Yan, F. Zhu, J. Wang, G. Cai, O. G. Schmidt, *Adv Funct Mater* **2024**.

[57] C. Liu, Y. Wang, Z. Qian, K. Wang, F. Zhao, P. Ding, D. Xu, G. Wei, L. Ren, L. Ren, *iScience* **2021**, *24*.

[58] C. Xu, Z. Jiang, T. Zhong, C. Chen, W. Ren, T. Sun, F. Fu, *ACS Omega* **2022**.

[59] W. Li, C. Lou, S. Liu, Q. Ma, G. Liao, K. C. F. Leung, X. Gong, H. Ma, S. Xuan, *Adv Funct Mater* **2024**.

[60] L. Ren, D. Wu, X. Ma, J. Li, J. Zhang, X. Zhang, Y. Yu, P. Xue, P. Lv, Y. Shao, P. Ma, Q. Wei, *Small* **2025**.

[61] M. Kalulu, O. Munyati, O. Oderinde, J. Hu, S. O. Ogungbesan, G. Fu, *J Appl Polym Sci* **2025**.

[62] X. Zhang, S. Aziz, B. Salahuddin, Z. Zhu, *ACS Appl Mater Interfaces* **2024**.

[63] Y. Zhang, B. Yuan, Y. Shi, X. Chen, Z. Wang, L. He, B. Wang, J. Xiao, M. Yu, Y. Gao, L. Zhang, C. Zou, R. Lan, H. Yang, *Mater Horiz* **2024**.

[64] Q. He, Z. Wang, Y. Wang, A. Minori, M. T. Tolley, S. Cai, *Sci Adv* **2019**, *5*.

[65] W. Jiang, G. Ye, B. Chen, H. Liu, *Soft Matter* **2021**, *17*, 8651.

[66] T. Yang, Z. Dong, C. Chen, J. Song, P. Ma, *Mater Des* **2025**, *252*.

[67] M. Kanik, S. Orguc, G. Varnavides, J. Kim, T. Benavides, D. Gonzalez, T. Akintilo, C. C. Tasan, A. P. Chandrakasan, Y. Fink, P. Anikeeva, *Science (1979)* **2019**, *365*, 145.

[68] W. Fan, C. Shan, H. Guo, J. Sang, R. Wang, R. Zheng, K. Sui, Z. Nie, *Dual-gradient enabled ultrafast biomimetic snapping of hydrogel materials*, **2019**.

[69] J. Qin, P. Feng, Y. Wang, X. Du, B. Song, *ACS Appl Mater Interfaces* **2020**, *12*, 46719.

[70] P. Jiang, Y. Zhang, X. Mu, D. Liu, Y. Liu, R. Guo, Z. Ji, X. Wang, X. Wang, *Adv Mater Technol* **2022**, *7*.

[71] H. Liu, X. Jia, R. Liu, K. Chen, Z. Wang, T. Lyu, X. Cui, Y. Zhao, Y. Tian, *J Mater Chem A Mater* **2022**, *10*, 21874.

[72] H. Le Ferrand, A. R. Studart, A. F. Arrieta, *ACS Nano* **2019**, *13*, 4752.

[73] J. U. Schmied, H. Le Ferrand, P. Ermanni, A. R. Studart, A. F. Arrieta, *Bioinspir Biomim* **2017**, *12*.

[74] S. Puthanveetil, W. C. Liu, K. S. Riley, A. F. Arrieta, H. Le Ferrand, *Compos Sci Technol* **2022**, *217*.

[75] K. Mo, J. Lin, P. Wei, J. Mei, C. Chang, *J Mater Chem C Mater* **2021**, *9*, 10295.

[76] K. Kim, Y. Guo, J. Bae, S. Choi, H. Y. Song, S. Park, K. Hyun, S. K. Ahn, *Small* **2021**, *17*.

[77] M. Ma, L. Guo, D. G. Anderson, R. Langer, *Science (1979)* **2013**, *339*, 186.

[78] M. Dai, O. T. Picot, J. M. N. Verjans, L. T. De Haan, A. P. H. J. Schenning, T. Peijs, C. W. M. Bastiaansen, *ACS Appl Mater Interfaces* **2013**, *5*, 4945.

[79] Q. Zhu, Y. Jin, W. Wang, G. Sun, D. Wang, *ACS Appl Mater Interfaces* **2019**, *11*, 1440.

[80] A. Ryabchun, F. Lancia, A. D. Nguindjel, N. Katsonis, *Soft Matter* **2017**, *13*, 8070.

[81] A. Sydney Gladman, E. A. Matsumoto, R. G. Nuzzo, L. Mahadevan, J. A. Lewis, *Nat Mater* **2016**, *15*, 413.

[82] Y. Ge, J. Zeng, B. Hu, D.-Y. Yang, Y. Shao, H. Lu, *Giant* **2022**, *11*, 100107.

[83] J. W. Boley, W. M. Van Rees, C. Lissandrello, M. N. Horenstein, R. L. Truby, A. Kotikian, J. A. Lewis, L. Mahadevan, *Proc Natl Acad Sci U S A* **2019**, *116*, 20856.

[84] X. Li, J. Liu, D. Li, S. Huang, K. Huang, X. Zhang, *Advanced Science* **2021**, *8*.

[85] X. Li, X. Cai, Y. Gao, M. J. Serpe, *J Mater Chem B* **2017**, *5*, 2804.

[86] Y. Jian, B. Wu, X. Le, Y. Liang, Y. Zhang, D. Zhang, L. Zhang, W. Lu, J. Zhang, T. Chen, *Research* **2019**, *2019*.

[87] H. Cui, N. Pan, W. Fan, C. Liu, Y. Li, Y. Xia, K. Sui, *Adv Funct Mater* **2019**, *29*.

[88] C. Ma, X. Le, X. Tang, J. He, P. Xiao, J. Zheng, H. Xiao, W. Lu, J. Zhang, Y. Huang, T. Chen, *Adv Funct Mater* **2016**, *26*, 8670.

[89] Y. Zhang, J. Liao, T. Wang, W. Sun, Z. Tong, *Adv Funct Mater* **2018**, *28*.

[90] P. Dong, W. Xu, Z. Kuang, Y. Yao, Z. Zhang, D. Guo, H. Wu, T. Zhao, A. Liu, *Advanced Intelligent Systems* **2021**, *3*.

[91] Q. L. Zhu, C. F. Dai, D. Wagner, O. Khoruzhenko, W. Hong, J. Breu, Q. Zheng, Z. L. Wu, *Advanced Science* **2021**, *8*.

[92] J. Hu, Z. Y. Kuang, L. Tao, Y. F. Huang, Q. Wang, H. Lou Xie, J. R. Yin, E. Q. Chen, *ACS Appl Mater Interfaces* **2019**, *11*, 48393.

[93] Y. Yang, T. Wang, F. Tian, X. Wang, Y. Hu, X. Xia, S. Xu, *Macromol Rapid Commun* **2021**, *42*.

[94] C. Zhang, X. Lu, G. Fei, Z. Wang, H. Xia, Y. Zhao, *ACS Appl Mater Interfaces* **2019**, *11*, 44774.

[95] Y. Wang, M. Li, J. K. Chang, D. Aurelio, W. Li, B. J. Kim, J. H. Kim, M. Liscidini, J. A. Rogers, F. G. Omenetto, *Nat Commun* **2021**, *12*.

[96] H. Tian, Z. Wang, Y. Chen, J. Shao, T. Gao, S. Cai, *ACS Appl Mater Interfaces* **2018**, *10*, 8307.

[97] H. Kim, J. A. Lee, C. P. Ambulo, H. B. Lee, S. H. Kim, V. V Naik, C. S. Haines, A. E. Aliev, R. Ovalle-Robles, R. H. Baughman, T. H. Ware, *Adv Funct Mater* **2019**, *29*.

[98] P. Xue, H. K. Bisoyi, Y. Chen, H. Zeng, J. Yang, X. Yang, P. Lv, X. Zhang, A. Priimagi, L. Wang, X. Xu, Q. Li, *Angewandte Chemie - International Edition* **2021**, *60*, 3390.

[99] Q. Zhao, Y. Chang, Z. Yu, Y. Liang, L. Ren, L. Ren, *J Mater Chem B* **2020**, *8*, 9362.

[100] S. Iamsaard, S. J. Aßhoff, B. Matt, T. Kudernac, J. J. L. M. Cornelissen, S. P. Fletcher, N. Katsonis, *Nat Chem* **2014**, *6*, 229.
